# Preventing suicide with Safe Alternatives for Teens and Youths (SAFETY): a randomised feasibility trial

**DOI:** 10.1136/bmjment-2025-301575

**Published:** 2025-04-29

**Authors:** Moa Karemyr, Martin Bellander, Moa Pontén, Anna Ohlis, Oskar Flygare, Ylva Walldén, Ralf Kuja-Halkola, Gergö Hadlaczky, David Mataix-Cols, Joan Rosenbaum Asarnow, Clara Hellner, Jennifer L Hughes, Johan Bjureberg

**Affiliations:** 1 Department of Clinical Neuroscience, Karolinska Institutet, Stockholm, Sweden; 2 Department of Global Public Health, Karolinska Institutet, Stockholm, Sweden; 3 Department of Women's and Children’s Health, Uppsala University, Uppsala, Sweden; 4 Department of Medical Epidemiology and Biostatistics, Karolinska Institutet, Stockholm, Sweden; 5 Department of Learning Management and Ethics, Karolinska Institutet, Stockholm, Sweden; 6 Department of Clinical Sciences, Lund University, Lund, Sweden; 7 Department of Psychiatry, University of California Los Angeles, Los Angeles, California, USA; 8 Department of Psychiatry and Behavioral Health, College of Medicine, and Division of Health Behavior and Health Promotion, College of Public Health, The Ohio State University, Columbus, Ohio, USA; 9 Big Lots Behavioral Health Services and Division of Child and Family Psychiatry, Nationwide Children’s Hospital, Columbus, Ohio, USA

**Keywords:** Child & adolescent psychiatry, Suicide & self-harm

## Abstract

**Background:**

Suicide attempts are common in youth and have potentially lethal outcomes. Effective treatments targeting suicide attempts are scarce.

**Objective:**

To assess the feasibility and preliminary efficacy of a family-based cognitive behavioural treatment relative to an active control for youth with suicidal behaviour.

**Methods:**

30 youths (93% female; mean (SD) age=14.6 (1.5) years) residing in Sweden with recent suicidal behaviour (last 3 months) and at least one available parent were randomised to 12 weeks of the family-based cognitive behavioural treatment Safe Alternatives for Teens and Youths (SAFETY) or supportive therapy, an active control treatment. Primary endpoint was 3-month post-treatment. Feasibility outcomes included treatment and assessment compliance, adverse events, treatment credibility and treatment satisfaction. Secondary outcomes included suicide attempt, non-suicidal self-injury, anxiety, depression, quality of life and emotion dysregulation.

**Findings:**

Both treatments showed high compliance, satisfaction, credibility and session completion, with few adverse events and dropouts as well as low attrition (7% at primary endpoint). At the primary endpoint, two (14%) participants in SAFETY and four (27%) in supportive therapy had attempted suicide. Non-suicidal self-injury was reduced by 95% (incidence rate ratio=0.05 (95% CI 0.01 to 0.20)) in SAFETY and 69% (incidence rate ratio=0.31 (0.11 to 0.83)) in supportive therapy. Participation in SAFETY, but not in supportive therapy, was associated with moderate-to-large within-group improvements in anxiety and depression (Cohen’s d=0.85 [0.33 to 1.40]), quality of life (d*=*1.01 [0.48 to 1.56]) and emotion dysregulation (d=1.22 [0.45 to 2.03]).

**Conclusions:**

The results suggest that SAFETY is feasible and promising for youth with suicidal behaviour.

**Clinical implications:**

A large randomised controlled trial is warranted to further examine the efficacy of SAFETY.

**Trial registration number:**

NCT05537623.

**WHAT IS ALREADY KNOWN ON THIS TOPIC**

- Suicide attempts are common among youths globally and associated with adverse and potentially lethal outcomes. Few have access to treatment targeting the risk of suicide reattempt.

**WHAT THIS STUDY ADDS**

- The findings of this study demonstrate the feasibility and acceptability of a family-based cognitive behavioural treatment named Safe Alternatives for Teens and Youths for youths with suicidal behaviour.

**HOW THIS STUDY MIGHT AFFECT RESEARCH, PRACTICE OR POLICY**

- The findings of this study require further evaluation in adequately powered randomised controlled trials.

## Background

 Suicide is a leading cause of death among 10–18 years old worldwide.^1^ One of the most robust predictors of future suicide attempt and suicide is prior suicide attempt.^2^ With a youth lifetime prevalence of 5–16%^3^ and many associated adverse outcomes,^4^ suicide attempts constitute an important target in intervention research.^5^

Dialectical behaviour therapy (DBT) remains the only established evidence-based clinical intervention targeting suicide attempt and non-suicidal self-injury (NSSI) among youths.^6^ Other treatments, such as brief cognitive behavioural therapies, family-based therapies and mentalisation-based therapies, have shown promising results in single trials.^5 7^ However, a recent individual participant data meta-analysis concluded that there is no evidence that therapeutic interventions were more effective than controls for reducing repeat self-harm in youth.^8^ While there is clearly a need for more research in this area, extensive work highlights the potential value of transdiagnostic treatment approaches targeting suicide attempts and NSSI independently of psychiatric comorbidity,^9^ as well as novel modes of treatment delivery, such as online^10^ or partially in-home^11^ delivery formats that can enhance availability and access.

Safe Alternatives for Teens and Youths (SAFETY)^11^ is a novel DBT-informed family-based ognitive behavioural therapy, delivered over 12 weeks, designed to target suicide attempts transdiagnostically in the heterogeneous high-risk group of youth presenting to the child and adolescent mental health services (CAMHS). Using mechanism-informed treatment approaches, SAFETY incorporates skills training and other interventions targeting transdiagnostic mechanisms, such as emotion regulation and connectedness.^9 10 12^ Participation in SAFETY has been linked to effects on suicide attempts, depression and hopelessness in two small trials (one open pilot study^13^ and one randomised controlled trial (RCT)^11^) with sample sizes of 35 and 42.

To our knowledge, SAFETY has not been evaluated outside the USA. More research, ideally conducted in a broader range of settings, is needed to clarify the feasibility and efficacy of SAFETY.

## Objective

The overall objective of this feasibility RCT was to lay the groundwork for a full-scale RCT by assessing the feasibility of SAFETY, relative to supportive therapy, for youth with suicidal behaviour. In addition, we aimed to provide preliminary data on the efficacy of SAFETY in Sweden.

## Methods

### Design

This study was a single-masked, parallel group feasibility RCT, comparing 12 weeks of SAFETY with supportive therapy for youths with suicidal behaviour and their parents. Participants were recruited within the CAMHS in Stockholm, Sweden, between 2 December 2022 and 10 November 2023. Primary endpoint was 3-month post-treatment. The study was registered at ClinicalTrials.gov (Identifier: NCT05537623). To ensure enhanced participant safety, a Data Safety and Monitoring Board led by an experienced child and adolescent psychiatrist (external from the research group) reviewed the study protocol before trial initiation and continuously during the trial. See online supplemental file 1 for the study protocol, including updates (approved by the Swedish Ethical Review Authority) and version history.

### Participants

Participants were referred from within CAMHS. Inclusion criteria were: (1) suicidal behaviour, defined as suicide attempt, interrupted/aborted suicide attempt or preparatory actions in the previous 3 months; (2) age 10–17 years; and (3) at least one primary caregiver willing to participate in treatment. Exclusion criteria were: (4) symptoms requiring other immediate treatment (eg, psychosis); (5) individual or life circumstances obstructing treatment (eg, in emergency foster care); and (6) insufficient understanding of the Swedish language.

### Procedures

The recruitment procedure included a face-to-face assessment at the clinic where a psychologist/social worker administered semistructured clinical interviews (eMethods). Participants and parents also completed self-rated assessments. Eligible participants were randomised in a 1:1 ratio to SAFETY or supportive therapy. Randomisation was conducted by an independent researcher, using a randomisation tool (www.random.org) to create a random sequence that was put in sealed opaque envelopes. The nine therapists were licensed clinical psychologists and a social worker, all trained in cognitive behavioural therapy. All therapists received training in and delivered both interventions to minimise potential therapist effects. Masked assessments and self-rated assessments were administered at post-treatment and 3-month post-treatment. Masked assessors were clinicians, external to the research team. See online supplemental file 2 and online supplemental table S1 for information on measures and assessment points, as well as on additional administered measures not included in this report.

### Feasibility outcomes

The feasibility outcomes included (1) treatment compliance, defined as the proportion of participants who received 6 or more and 12 or more treatment sessions, respectively; (2) assessment compliance, defined as the proportion of participants who completed primary endpoint assessments; (3) study-related adverse events, evaluated by a child and adolescent psychiatrist following Good Clinical Practice; (4) treatment credibility (Credibility/Expectancy Questionnaire),^14^ and (5) treatment satisfaction (Client Satisfaction Questionnaire).^15^

### Exploratory secondary outcomes

#### Clinical outcomes

Clinical outcomes included suicide attempt (Columbia Suicide Severity Rating Scale; C-SSRS); NSSI (Deliberate Self-Harm Inventory–Youth Version; DSHI-Y); symptom severity and improvement (Clinical Global Impression–Severity; CGI-S and Clinical Global Impression–Improvement); global function (Children’s Global Assessment Scale; CGAS), all administered by a masked assessor. Additional clinical outcomes were youth-rated and included functional impairment (Work and Social Adjustment Scale–Youth version; WSAS-Y); health-related quality of life (Child Health Utility–9D; CHU-9D); depression and anxiety (Revised Children’s Anxiety and Depression Scale; RCADS); and self-destructive behaviours (Borderline Symptom List–Behaviour Supplement; BSL-Supplement). See online supplemental file 2 and online supplemental table S1 for references and descriptions of these measures.

#### Target mechanisms

The target mechanisms were youth-rated and included emotion dysregulation (Difficulties in Emotion Regulation Scale–16 item-version), and hopelessness (Beck’s Hopelessness Scale; BHS). See online supplemental file 2 and online supplemental table S1 for references and descriptions of these measures.

#### Parental outcomes

Parental outcomes were parent-rated and included depression (Patient Health Questionnaire–9); anxiety (Generalised Anxiety Disorder 7-item Scale); hopelessness, assessed with BHS; parental coping with their children’s emotions (Coping with Children’s Negative Emotions Scale; CCNES); and youth functional impairment (WSAS-Y). See online supplemental file 2 and online supplemental table S1 for references and descriptions of these measures.

### Interventions

SAFETY^11^ is a DBT-informed transdiagnostic family-based cognitive behavioural therapy. The 12-week programme includes weekly sessions with two therapists dedicated to each family. SAFETY is structured in phases and individually tailored based on a cognitive-behavioural fit analysis that specifies key risk and protective processes related to suicidal behaviour within the youth, the family and the youth’s environment. The individual tailoring results in a flexible treatment plan incorporating interventions from DBT as well as other forms of evidence-based treatments as needed. Each session includes an individual component with the youth and parents, respectively, and one family component where youth and parents work together with therapists to practice skills identified as critical for preventing future suicide attempts. Treatment targets are based on the fit analysis for each family. These frequently include strengthening protective support and validation, while using skills training with both youth and parents to increase adaptive strategies to cope with painful emotions and stress such as emotion regulation, problem-solving, self-validation and activity scheduling. To aid the skill acquisition process, therapists are available for skills coaching by telephone during office hours. Online supplemental table S2 provides an overview of the treatment.

Supportive therapy^6^ is a manualised client-centred therapy, adapted to match SAFETY regarding treatment dosage to control for non-specific treatment factors such as therapist attention and characteristics, time and treatment exposure. The supportive therapy used in this trial was modelled after that used successfully in the US DBT trial with highly suicidal self-harming youths.^6^ Supportive therapy is primarily an individual treatment for the youth, focusing on the supporting relationship between the therapist and the youth. Parental contact includes weekly follow-ups over the phone, as well as individual sessions (minimum 1 monthly parent session). Family sessions are only allowed when necessary to care for the youth’s safety, to aid crisis planning and assessment of imminent suicide risk. Therapeutic strategies include validation and attending to emotions.

Other ongoing treatment was allowed in both conditions for ethical reasons and was monitored throughout the trial.

#### Therapist treatment Fidelity

The therapists received supervision from psychologists specialising in the respective treatments, with sessions scheduled 1–2 times per month. All treatment sessions were videotaped. Supervisors and therapists reviewed the videos together to address challenges, with supervisors monitoring fidelity. A randomly selected sample of 10% of SAFETY sessions was rated for fidelity, indicating strong adherence (see online supplemental file 2).

### Statistical analysis

Following recommendations from the Consort 2010 Statement extension for feasibility RCTs,^16^ the sample size of 30 participants was deemed suitable for assessing feasibility in this RCT, while the trial was not powered for between-group effects. Descriptive statistics were calculated for baseline characteristics, feasibility outcomes and suicide attempts (C-SSRS). Secondary within-group analyses were conducted according to the intention-to-treat principle, including all participants as randomised. For count outcomes, two different models were selected based on Akaike information criterion (AIC) and Bayesian information criterion (BIC) scores (see online supplemental table S3 for model comparisons). Zero-inflated negative binomial generalised linear-mixed effects regression tested within-group effects on NSSI (DSHI-Y), and zero-inflated Poisson tested within-group effects on self-destructive behaviours (BSL-Supplement). Effect sizes are presented as incidence rate ratios (IRR) with CIs between time-points for the separate conditions. Ordinal outcomes were analysed with linear quantile mixed-effect regression.^17^ Effect sizes were calculated by dividing the unstandardised betas with the IQR at the first time point, and bootstrap (1000 simulations) CI were calculated.^17^ For continuous outcomes, linear mixed effects regressions were fitted. Effect sizes were evaluated with Cohen’s d for mixed effects models (calculated by dividing the unstandardised betas by the SD at the first time point) with bootstrap (1000 simulations) CI. All models were fitted separately for each treatment condition and included a random intercept and the dummy coded time variable (pretreatment, post-treatment and 3-month post-treatment). Mixed-effect regressions for repeated measures are valid under the assumption that the data are missing at random,^18^ which was assumed in the current study. Masked assessors’ treatment allocation guesses were analysed with a binomial test. Statistical analyses were conducted using R (V.4.3.1). All tests were two-sided and statistical significance was set at p<0.05.

## Findings

**Figure 1:**
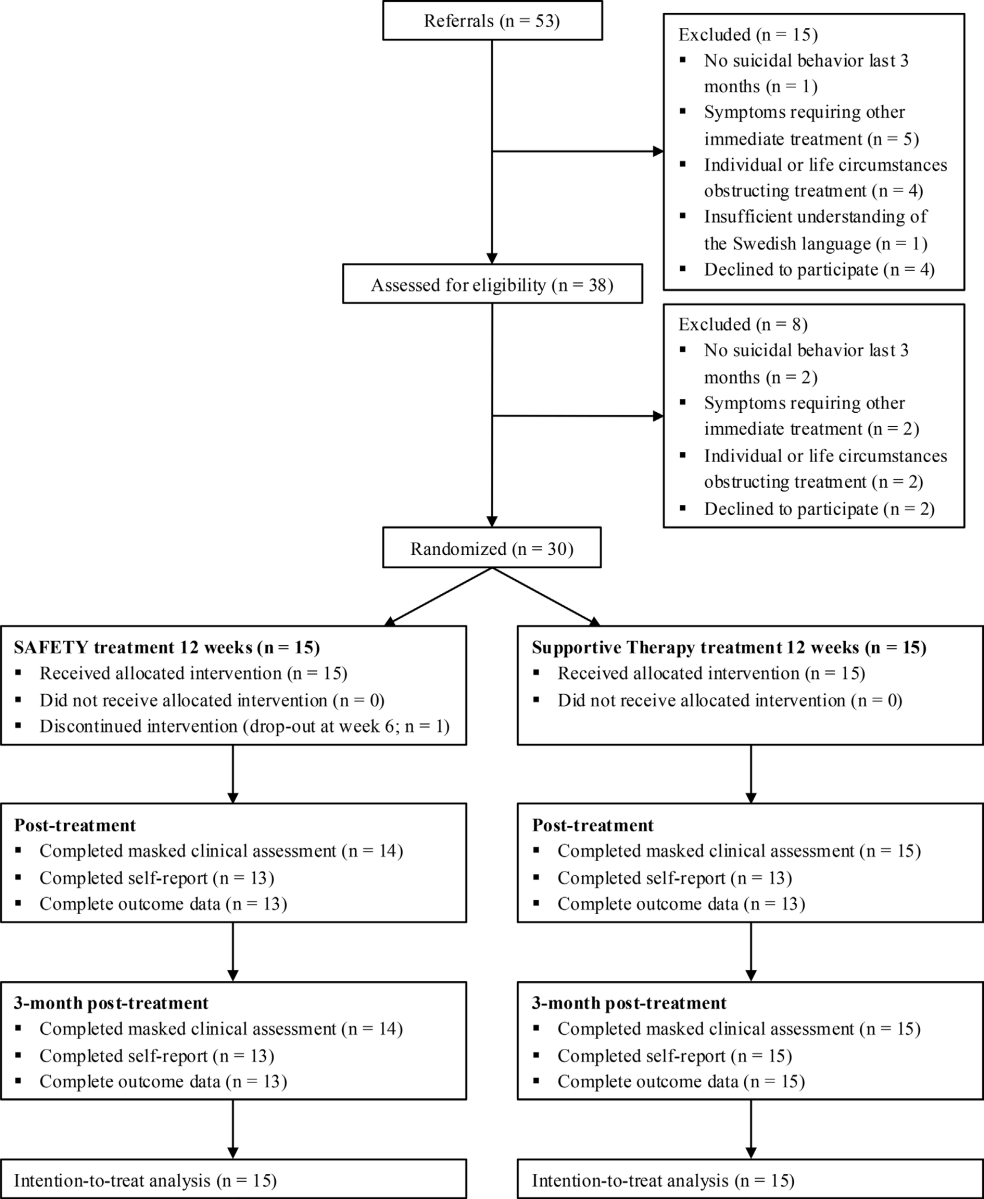
Flowchart of participant enrolment and study progression. SAFETY, Safe Alternatives for Teens and Youths.

Between December 2022 and November 2023, 30 participants (93% female) aged 11.6–17.6 (mean=14.6, SD=1.5) were recruited and randomised to either SAFETY (n=15) or supportive therapy (n=15). See figure 1 for flowchart and table 1 for participant characteristics.

**Table 1:** Study and participant characteristics

	No. (%)SAFETY(n=15)	No. (%)Supportive therapy(n=15)	No. (%)Total(n=30)
Study characteristics			
Gender			
Female	14 (93)	14 (93)	28 (93)
Male	1 (7)	0 (0)	1 (3)
Non-binary	0 (0)	1 (7)	1 (3)
Age in years, mean (SD)	14.6 (1.8)	14.6 (1.2)	14.6 (1.5)
Region of birth			
Sweden	14 (93)	13 (87)	27 (90)
Europe	0 (0)	2 (13)	2 (7)
Asia	1 (7)	0 (0)	1 (3)
Parent characteristics*			
Mother	11 (73)	10 (67)	21 (70)
Father	4 (27)	5 (33)	9 (30)
Highest level of education			
Primary school	1 (7)	1 (7)	2 (7)
Secondary school	3 (20)	4 (27)	7 (23)
College or university <3 years	2 (13)	0 (0)	2 (7)
College or university ≥3 years	8 (53)	9 (60)	17 (57)
Doctorate	1 (7)	1 (7)	2 (7)
Parent occupational status			
Employed or self-employed	14 (93)	15 (100)	29 (97)
Unemployed or on sick-leave	1 (7)	0 (0)	1 (3)
Regions of birth for both biological parents			
Sweden	22 (73)	19 (63)	41 (68)
Europe	2 (7)	5 (17)	7 (12)
Africa	1 (3)	4 (13)	5 (8)
Asia	1 (3)	2 (7)	3 (1)
South America	3 (1)	0 (0)	3 (1)
North America	0 (0)	1 (3)	1 (0)
Participant characteristics			
Suicidal behaviour, last 3 months			
Suicide attempt	11 (73)	13 (87)	24 (80)
Interrupted suicide attempt	1 (7)	2 (13)	3 (10)
Aborted suicide attempt	1 (7)	0 (0)	1 (3)
Preparatory actions	2 (13)	0 (0)	2 (7)
Individuals with lifetime suicide attempt	11 (73)	13 (87)	24 (80)
No. of lifetime suicide attempts, mean (SD)	1.7 (2.5)	1.1 (0.6)	1.4 (1.8)
Individuals with NSSI, last 3 months	15 (100)	11 (73)	26 (87)
Comorbidity†			
Major depressive disorder	11 (73)	10 (67)	21 (70)
Social anxiety disorder	10 (67)	6 (40)	16 (53)
Panic disorder/agoraphobia	6 (40)	4 (27)	10 (33)
Post-traumatic stress disorder	1 (7)	2 (13)	3 (10)
Generalised anxiety disorder	6 (40)	3 (20)	9 (30)
Separation anxiety disorder	3 (20)	0 (0)	3 (10)
Specific phobia disorder	5 (33)	6 (40)	11 (37)
OCD/BDD	2 (13)	1 (7)	3 (10)
Tics/Tourette’s syndrome	1 (7)	3 (20)	4 (13)
Eating disorder	2 (13)	0 (0)	2 (7)
ADHD, diagnosis‡§	6 (40)	6 (40)	12 (40)
ADHD, screened positive‡	12 (80)	9 (60)	21 (70)
Autism spectrum disorder§	3 (20)	3 (20)	6 (20)
Alcohol or substance dependence	1 (7)	1 (7)	2 (7)
Oppositional defiant disorder	2 (13)	0 (0)	2 (7)
No. of co-occurring disorders, mean (SD)	7.1 (3.1)	5.3 (1.9)	6.2 (2.7)
Fulfilling ≥5 BPD criteria	1 (7)	0 (0)	1 (3)
No. of BPD criteria, mean (SD)	2.5 (1.2)	2.1 (1.3)	2.2 (1.3)
Previous psychosocial treatment			
Ever received inpatient care	7 (47)	3 (20)	10 (33)
Previous counselling	11 (73)	11 (73)	22 (73)
Time in previous counselling, mean (SD) months	7.8 (15)	12.7 (14.5)	10.2 (14.7)
Ongoing counselling at inclusion	5 (33)	10 (67)	15 (50)
Time in ongoing counselling, mean (SD) months	1.4 (3.0)	3.9 (5.6)	2.8 (4.6)
Type of ongoing counselling			
Supportive therapy	3 (20)	3 (20)	6 (20)
Cognitive behavioural therapy	2 (13)	5 (33)	7 (23)
Other/do not know	10 (67)	7 (47)	17 (57)
Ongoing pharmacological medication at inclusion (ATC code)¶			
Any ongoing psychopharmacological medication	12 (80)	10 (67)	22 (73)
Antidepressants (N06A)			
SSRI (N06AB)	9 (60)	7 (47)	16 (53)
Other antidepressants (N06AX)	0 (0)	1 (7)	1 (3)
Hypnotics and sedatives (N05C)			
Melatonin receptor agonists (N05CH)	7 (47)	4 (27)	11 (37)
Other hypnotics and sedatives (N05CM)	2 (13)	0 (0)	2 (7)
Antihistamines for systemic use (R06A)	10 (67)	3 (20)	13 (43)
Psychostimulants (N06B)	2 (13)	3 (20)	5 (17)
Antipsychotics (N05A)	2 (13)	1 (7)	3 (10)

* Characteristics of the parent primarily involved in treatment and assessments.

† Assessed by the research team using the MINI-KID International Neuropsychiatric Interview, version 6.

‡ Includes both combined, primarily inattentive and primarily hyperactive-impulsive subtype.

§ Parent-reported registered diagnosis.

¶ Classes of medication were based on WHO anatomic therapeutic chemical categories.

ADHD, attention-deficit hyperactivity disorder; ATC, anatomical therapeutic chemical; BDD, body dysmorphic disorder; BPD, borderline personality disorder; NSSI, non-suicidal self-injury; OCD, obsessive-compulsive disorder; SAFETY, Safe Alternatives for Teens and Youths; SSRI, selective serotonin reuptake inhibitors.

### Feasibility outcomes

At post-treatment, 14 (93%) participants in each condition had received at least six treatment sessions. 11 (73%) participants in SAFETY and 9 (60%) participants in supportive therapy had received at least 12 sessions (ie, the full treatment).

Regarding assessment compliance, 28 (93%) participants had complete outcome data at 3-month post-treatment. 29 (97%) had data from masked assessments (of which 3 (10%) were conducted only with the parent and 1 (3%) was conducted with a non-masked assessor). 28 (93%) had complete self-rated data.

Study-related adverse events were reported twice in SAFETY: a self-rating assessment was perceived as prompting urges for NSSI, and one family expressed displeasure when social services became involved, which is standard procedure at local CAMHS after a suicide attempt.

Mean treatment credibility was 5.84 (SD=1.29) in SAFETY and 4.90 (SD=1.33) in supportive therapy, indicating satisfactory credibility. Treatment satisfaction was generally high in both conditions. In SAFETY, 12 (92%) participants and 13 (93%) parents rated treatment quality as good/excellent and would recommend it. In supportive therapy, 10 (77%) participants and 9 (60%) parents rated quality as good/excellent, with 11 (85%) participants and 10 (67%) parents recommending it.

### Secondary outcomes

#### Clinical outcomes

Between pretreatment and post-treatment, two (14%) participants in SAFETY and one (7%) participant in supportive therapy attempted suicide. At 3-month post-treatment, no additional participants in SAFETY and three additional (20%) participants in supportive therapy attempted suicide, leaving a total of two (14%) SAFETY participants and four (27%) supportive therapy participants having engaged in suicide attempts between pretreatment and 3-month post-treatment (primary endpoint). Significant reductions in NSSI were detected from pretreatment to 3-month post-treatment in both SAFETY (IRR=0.05, 95% CI 0.01 to 0.20) and supportive therapy (IRR=0.31, 95% CI 0.11 to 0.83).

In SAFETY, improvements in other self-destructive behaviours from pretreatment to 3-month post-treatment were non-significant (IRR=0.68, 95% CI 0.35 to 1.34), whereas supportive therapy showed significant reductions (IRR=0.38, 95% CI 0.22 to 0.67). Symptom severity decreased from pretreatment to 3-month post-treatment in SAFETY (CGI-S; effect size (medians relative to the IQR) = 0.86, 95% CI 0.21 to 1.51), but not in supportive therapy (effect size=0.30, 95% CI −0.31 to 0.92). Large improvements in global functioning were detected from pretreatment to 3-month post-treatment in both SAFETY (CGAS; d=1.52, 95% CI 0.92 to 2.13) and supportive therapy (d=1.62, 95% CI 0.66 to 2.59). SAFETY participants demonstrated moderate improvements in functional impairment from pretreatment to 3-month post-treatment, as rated by both parents (WSAS-Y; d=0.65, 95% CI 0.26 to 1.04) and youth (d=0.65, 95% CI 0.23 to 1.09), whereas non-significant improvements were found for supportive therapy, in both parent (d=0.18, 95% CI −0.26 to 0.63) and youth ratings (d=0.44, 95% CI −0.45 to 1.37). SAFETY participants showed large reductions in anxiety and depression from pretreatment to 3-month post-treatment (RCADS-25; d=0.85, 95% CI 0.33 to 1.40), whereas non-significant improvements were found in supportive therapy (d=0.67, 95% CI −0.06 to 1.42). In SAFETY, large improvements in health-related quality of life were observed from pretreatment to 3-month post-treatment (CHU-9D; d=1.01, 95% CI 0.48 to 1.56) but not in supportive therapy (d=0.25, 95% CI 0.46 to 0.98). See table 2 for results.

**Table 2:** Results for clinical outcomes

Count outcomes	Median (Q1, Q3)	n†	Fixed effects		Effect size‡	
			β (SE)	P value	IRR	95% CI
Masked assessor-rated NSSI (DSHI-Y)§						
SAFETY						
Pre-treatment	14 (9, 52.5)	15				
Post-treatment	3 (1, 15.8)	14	−1.59 (0.58)	0.006	0.20	0.05 to 0.84
3-M post	0 (0, 1)	13	−3.07 (0.65)	<0.001	0.05	0.01 to 0.20
Supportive therapy						
Pre-treatment	20 (0.5, 35.5)	15				
Post-treatment	3 (0, 10)	14	−0.74 (0.51)	0.149	0.48	0.20 to 1.17
3-M post	2 (0, 10)	13	−1.17 (0.53)	0.029	0.31	0.11 to 0.83
Self-rated self-destructive behaviours (BSL-Supplement)¶						
SAFETY						
Pre-treatment	3 (1.5, 5.5)	15				
Post-treatment	0 (0, 2)	13	−0.11 (0.36)	0.770	0.90	0.53 to 1.52
3-M post	0.5 (0, 2.5)	14	−0.38 (0.30)	0.205	0.68	0.35 to 1.34
Supportive therapy						
Pre-treatment	2 (0, 3.5)	15				
Post-treatment	0 (0, 1)	14	−1.17 (0.37)	0.001	0.31	0.17 to 0.57
3-M post	0 (0, 1)	15	−0.97 (0.34)	0.004	0.38	0.22 to 0.67
**Ordinal outcomes**	**Median (Q1, Q3)**	**n†**	**β (SE**)	**P value**	Effect size**	**95%CI**
Masked assessor-rated clinical global symptom severity (CGI-S)††						
SAFETY						
Pre-treatment	5 (4, 5)	15				
Post-treatment	4.5 (4, 5.75)	14	0.00 (0.27)	1	0.00	−0.53 to 0.53
3-M post	4 (4, 5)	14	−0.86 (0.33)	0.009	0.86	0.21 to 1.51
Supportive therapy						
Pre-treatment	5 (4, 5.75)	15				
Post-treatment	4 (4, 5)	15	−0.30 (0.28)	0.274	0.30	−0.24 to 0.85
3-M post	4 (3.5, 5)	15	−0.30 (0.32)	0.313	0.30	−0.31 to 0.92
Masked assessor-rated clinical global symptom improvement (CGI-I)‡‡						
SAFETY						
Post-treatment	3 (2, 3)	14	NA	NA	NA	NA
3-M post	2.5 (2, 3)	14	NA	NA	NA	NA
Supportive therapy						
Post-treatment	3 (3, 3)	15	NA	NA	NA	NA
3-M post	3 (3, 3.5)	15	NA	NA	NA	NA
**Continuous outcomes**	**Mean (SD)**	**n†**	**β (SE**)	**P value**	**d**	**95%CI**
Masked assessor-rated global functioning (CGAS)§§						
SAFETY						
Pre-treatment	43.00 (6.12)	15				
Post-treatment	52.00 (9.50)	14	8.65 (1.92)	<0.001	1.41	0.79 to 2.03
3-M post	52.64 (8.62)	14	9.29 (1.92)	<0.001	1.52	0.92 to 2.13
Supportive therapy						
Pre-treatment	45.00 (5.72)	15				
Post-treatment	53.13 (15.10)	15	8.13 (2.79)	0.007	1.42	0.48 to 2.37
3-M post	54.27 (14.46)	15	9.27 (2.79)	0.003	1.62	0.66 to 2.59
Self-rated functional impairment (WSAS)§§						
SAFETY						
Pre-treatment	22.33 (8.07)	15				
Post-treatment	21.23 (9.41)	13	−1.55 (1.86)	0.413	0.19	−0.26 to 0.65
3-M post	17.79 (7.14)	14	−5.23 (1.81)	0.008	0.65	0.23 to 1.09
Supportive therapy						
Pre-treatment	24.53 (5.73)	15				
Post-treatment	22.69 (10.55)	13	−2.06 (2.71)	0.455	0.36	−0.56 to 1.32
3-M post	22.00 (9.55)	15	−2.53 (2.59)	0.337	0.44	−0.45 to 1.37
Self-rated anxiety and depression (RCADS-25)§§						
*Anxiety subscale*						
SAFETY						
Pre-treatment	21.80 (7.03)	15				
Post-treatment	18.62 (6.10)	13	−3.25 (1.78)	0.079	0.46	−0.04 to 0.96
3-M post	17.57 (6.09)	14	−4.26 (1.73)	0.021	0.61	0.14 to 1.09
Supportive therapy						
Pre-treatment	17.47 (5.44)	15				
Post-treatment	17.38 (3.88)	13	−0.08 (1.67)	0.961	0.02	−0.58 to 0.64
3-M post	15.07 (3.61)	15	−2.40 (1.61)	0.144	0.44	−0.15 to 1.05
*Depression subscale*						
SAFETY						
Pre-treatment	20.13 (6.19)	15				
Post-treatment	15.85 (3.93)	13	−4.70 (1.69)	0.011	0.76	0.22 to 1.30
3-M post	15.43 (5.49)	14	−5.08 (1.65)	0.006	0.82	0.32 to 1.34
Supportive therapy						
Pre-treatment	18.53 (3.16)	15				
Post-treatment	16.31 (5.02)	13	−2.26 (1.43)	0.126	0.71	−0.17 to 1.63
3-M post	15.80 (5.20)	15	−2.73 (1.37)	0.056	0.87	0.01 to 1.75
*Total score*						
SAFETY						
Pre-treatment	41.93 (10.91)	15				
Post-treatment	34.46 (8.23)	13	−7.89 (3.11)	0.017	0.72	0.16 to 1.28
3-M post	33.00 (9.92)	14	−9.30 (3.03)	0.005	0.85	0.33 to 1.40
Supportive therapy						
Pre-treatment	36.00 (7.65)	15				
Post-treatment	33.69 (7.32)	13	−2.32 (2.91)	0.433	0.30	−0.44 to 1.07
3-M post	30.87 (8.11)	15	−5.13 (2.81)	0.079	0.67	−0.06 to 1.42
Self-rated health-related quality of life (CHU-9D)§§						
SAFETY						
Pre-treatment	19.40 (6.76)	15				
Post-treatment	16.23 (6.99)	13	−3.31 (1.93)	0.097	0.49	−0.07 to 1.05
3-M post	12.79 (5.85)	14	−6.83 (1.88)	0.001	1.01	0.48 to 1.56
Supportive therapy						
Pre-treatment	17.27 (5.38)	15				
Post-treatment	16.15 (8.23)	13	−1.12 (2.00)	0.580	0.21	−0.52 to 0.96
3-M post	15.93 (6.55)	15	−1.33 (1.91)	0.492	0.25	−0.46 to 0.98

* Of count, ordinal and continuous outcomes evaluating change from pre-treatment to post-treatment and 3-month post-treatment (primary endpoint). Fixed-effects parameter estimates β (SE) represent the effect of time with all randomised individuals (n=30). Medians (Q1s and Q3s) and means (SDs) are observed values.

† Numbers of participants contributing with data.

‡ Positive effect size indicate improvement between time points.

§ This outcome was analysed using a zero-inflated negative binomial regression model.

¶ This outcome was analysed using a zero-inflated Poisson regression model.

** Medians relative to the IQR.

†† This outcome was analysed using a linear quantile mixed-effect regression model.

‡‡ Due to the nature of this measure, only observed values are presented. Thus, fixed effects and effect sizes are presented as NAs.

§§ This outcome was analysed using a linear mixed effects regression model assuming a normal distribution.

BSL, Borderline Symptom List; CGAS, Children’s Global Assessment Scale; CGI-I, Clinical global impression–Improvement; CGI-S, Clinical global impression–Severity; CHU-9D, Child Health Utility–9D; d, Cohen’s d; DSHI-Y, Deliberate Self-Harm Inventory–Youth Version; IRR, incidence rate ratio; 3-M post, 3-month post-treatment; NA, not applicable; NSSI, non-suicidal self-injury; Q, Quartile; RCADS-25, The Revised Children’s Anxiety and Depression Scale-25; SAFETY, Safe Alternatives for Teens and Youths; WSAS, Work and Social Adjustment Scale.

#### Target mechanisms

Large improvements in emotion dysregulation were observed from pretreatment to 3-month post-treatment in SAFETY (d=1.22, 95% CI 0.45 to 2.03), but not in supportive therapy (d=0.60, 95% CI 0.01 to 1.22). Large improvements in hopelessness were detected in both SAFETY (d=1.23, 95% CI 0.65to 1.88) and supportive therapy (d=0.90, 95% CI 0.14 to 1.66). See table 3 for results.

**Table 3:** Results for target mechanisms and parental outcomes*

Continuous outcomes	Mean (SD)	n†	Fixed effects		Effect size‡	
			β (SE)	P value	d	95% CI
**Target mechanisms**						
Self-rated difficulties in emotion regulation (DERS-16)§						
SAFETY						
Pre-treatment	61.73 (11.60)	15				
Post-treatment	48.92 (15.96)	13	−12.74 (4.86)	0.014	1.10	0.27 to 1.92
3-M post	47.36 (14.73)	14	−14.19 (4.75)	0.006	1.22	0.45 to 2.03
Supportive therapy						
Pre-treatment	58.73 (11.25)	15				
Post-treatment	57.23 (9.28)	13	−2.21 (3.52)	0.536	0.20	−0.41 to 0.83
3-M post	51.93 (12.81)	15	−6.80 (3.36)	0.054	0.60	0.01 to 1.22
Self-rated hopelessness (BHS)§						
SAFETY						
Pre-treatment	14.87 (5.17)	15				
Post-treatment	11.38 (7.37)	13	−3.86 (1.70)	0.032	0.75	0.10 to 1.39
3-M post	8.93 (8.01)	14	−6.50 (1.65)	0.001	1.26	0.65 to 1.88
Supportive therapy						
Pre-treatment	15.07 (4.23)	15				
Post-treatment	11.71 (6.46)	14	−3.50 (1.60)	0.037	0.83	0.08 to 1.58
3-M post	11.27 (7.19)	15	−3.80 (1.56)	0.022	0.90	0.14 to 1.66
**Parental outcomes**						
Parent-rated depressive symptoms (PHQ-9)§						
SAFETY						
Pre-treatment	6.87 (4.94)	15				
Post-treatment	6.57 (6.11)	14	−0.19 (1.17)	0.871	0.04	−0.43 to 0.51
3-M post	6.36 (5.43)	14	−0.41 (1.17)	0.731	0.08	−0.38 to 0.54
Supportive therapy						
Pre-treatment	6.47 (5.76)	15				
Post-treatment	4.53 (3.66)	15	−1.93 (1.34)	0.161	0.34	−0.12 to 0.79
3-M post	5.87 (4.85)	15	−0.60 (1.34)	0.659	0.10	−0.36 to 0.56
Parent-rated anxiety symptoms (GAD-7)§						
SAFETY						
Pre-treatment	7.47 (5.44)	15				
Post-treatment	5.64 (4.41)	14	−1.55 (1.32)	0.252	0.29	−0.19 to 0.77
3-M post	4.71 (4.12)	14	−2.48 (1.32)	0.073	0.46	−0.02 to 0.92
Supportive therapy						
Pre-treatment	7.67 (5.74)	15				
Post-treatment	4.67 (4.43)	15	−3.00 (1.25)	0.023	0.52	0.10 to 0.94
3-M post	5.53 (4.39)	15	−2.13 (1.25)	0.098	0.37	−0.06 to 0.80
Parent-rated hopelessness (BHS)§						
SAFETY						
Pre-treatment	4.20 (4.57)	15				
Post-treatment	4.36 (3.39)	14	0.04 (0.89)	0.960	−0.01	−0.39 to 0.38
3-M post	4.79 (5.21)	14	0.47 (0.89)	0.598	−0.10	−0.48 to 0.27
Supportive therapy						
Pre-treatment	2.47 (2.20)	15				
Post-treatment	1.87 (2.77)	15	−0.60 (0.75)	0.431	0.27	−0.39 to 0.93
3-M post	3.07 (4.45)	15	0.60 (0.75)	0.431	−0.27	−0.95 to 0.40
Parent-rated coping with children’s negative emotions (CCNES)§						
*Expressive encouragement subscale*						
SAFETY						
Pre-treatment	5.64 (0.75)	15				
Post-treatment	5.79 (0.71)	14	0.17 (0.21)	0.442	0.22	−0.34 to 0.78
3-M post	5.52 (0.98)	14	−0.11 (0.21)	0.606	−0.15	−0.69 to 0.40
Parent-rated coping with children’s negative emotions (CCNES)§						
*Expressive encouragement subscale*						
Supportive therapy						
Pre-treatment	5.50 (0.81)	15				
Post-treatment	5.75 (0.67)	15	0.24 (0.19)	0.212	0.30	−0.15 to 0.76
3-M post	5.79 (0.67)	15	0.28 (0.19)	0.152	0.35	−0.12 to 0.81
*Minimisation subscale*						
SAFETY						
Pre-treatment	2.86 (0.93)	15				
Post-treatment	1.94 (0.91)	14	−0.90 (0.17)	<0.001	0.96	0.59 to 1.33
3-M post	2.08 (0.83)	14	−0.76 (0.17)	<0.001	0.82	0.45 to 1.18
Supportive therapy						
Pre-treatment	2.66 (0.81)	15				
Post-treatment	2.07 (0.65)	15	−0.59 (0.16)	0.001	0.72	0.33 to 1.11
3-M post	2.08 (0.79)	15	−0.58 (0.16)	0.001	0.71	0.31 to 1.11
*Punitive reactions subscale*						
SAFETY						
Pre-treatment	1.61 (0.67)	15				
Post-treatment	1.40 (0.54)	14	−0.24 (0.12)	0.045	0.36	0.02 to 0.70
3-M post	1.53 (0.61)	14	−0.11 (0.12)	0.357	0.16	−0.17 to 0.50
Supportive therapy						
Pre-treatment	1.51 (0.39)	15				
Post-treatment	1.31 (0.37)	15	−0.20 (0.11)	0.073	0.51	−0.02 to 1.05
3-M post	1.39 (0.44)	15	−0.12 (0.11)	0.279	0.31	−0.24 to 0.85
*Distress subscale*						
SAFETY						
Pre-treatment	2.04 (1.08)	15				
Post-treatment	1.67 (0.71)	14	−0.36 (0.18)	0.056	0.33	0.01 to 0.67
3-M post	1.77 (0.73)	14	−0.27 (0.18)	0.152	0.25	−0.08 to 0.57
Supportive therapy						
Pre-treatment	1.80 (0.59)	15				
Post-treatment	1.75 (0.64)	15	−0.05 (0.19)	0.787	0.09	−0.54 to 0.71
3-M post	1.73 (0.82)	15	−0.07 (0.19)	0.700	0.13	−0.52 to 0.76
Parent-rated youth functional impairment (WSAS)§						
SAFETY						
Pre-treatment	24.67 (10.04)	15				
Post-treatment	22.86 (8.67)	14	−1.94 (2.03)	0.349	0.19	−0.21 to 0.60
3-M post	18.29 (10.69)	14	−6.51 (2.03)	0.004	0.65	0.26 to 1.04
Supportive therapy						
Pre-treatment	18.13 (9.18)	15				
Post-treatment	21.00 (10.64)	15	2.87 (2.06)	0.176	0.31	−0.12 to 0.75
3-M post	19.80 (10.14)	15	1.67 (2.06)	0.426	0.18	−0.26 to 0.63

* Of continuous outcomes evaluating change from pre-treatment to post-treatment and 3-month post-treatment (primary endpoint). Fixed-effects parameter estimates β (SE) represent the effect of time with all randomised individuals (n=30). Means (SDs) are observed values.

† Numbers of participants contributing with data.

‡ Positive effect size indicate improvement between time points.

§ This outcome was analysed using a linear mixed effects regression model assuming a normal distribution.

BHS, Beck’s Hopelessness Scale; CCNES, Coping with Children’s Negative Emotion Scale; d, Cohen’s d; DERS-16, Difficulties in Emotion Regulation Scale–Brief version; GAD-7, Generalised Anxiety Disorder-7 item scale; 3-M post, 3-month post-treatment; PHQ-9, Patient Health Questionnaire-9; SAFETY, Safe Alternatives for Teens and Youths; WSAS, Work and Social Adjustment Scale.

#### Parental outcomes

Large improvements were detected in minimisation of children’s negative emotions (ie, dismissing the child’s emotional responses), from pretreatment to 3-month post-treatment in parents in SAFETY (CCNES; d=0.82, 95% CI 0.45 to 1.18), while moderate improvements were detected in parents in supportive therapy (d=0.71, 95% CI 0.31 to 1.11). For remaining parental outcomes, see table 3.

#### Inter-rater reliability

A random 20% of masked suicide attempt assessments at post-treatment and 3-month post-treatment were rerated, showing 100% inter-rater reliability.

#### Masking integrity

All but one clinical assessment conducted at post-treatment and 3-month post-treatment were conducted by masked assessors with intact masking integrity. Masked assessors were not better than chance at guessing treatment allocation at either post-treatment (proportion correct=0.62, 95% CI 0.21 to 0.56, p=0.265) or at 3-month post-treatment (proportion correct=0.52, 95% CI 0.32 to 0.71, p=1).

#### Additional treatments

Additional psychopharmacological and psychosocial treatment received during trial participation were comparable across conditions and are described in online supplemental tables S5 and S6, available online.

## Discussion

Results from this RCT support the feasibility of evaluating SAFETY for youth with suicidal behaviour. Compliance to treatment and assessments was high, as were treatment satisfaction and credibility. Few study-related adverse events were reported. Two participants in SAFETY and four participants in supportive therapy attempted suicide during study participation. Only supportive therapy participants (n=3) attempted suicide between post-treatment and 3-month post-treatment. The proportions of suicide attempts in SAFETY versus active control in this study were similar to those reported in the previous US trial of SAFETY.^11^ Participants in both conditions experienced reductions in NSSI, improved global function and decreased hopelessness, while SAFETY participants also experienced decreased symptom severity and functional impairment, as well as improvements in anxiety, depression, health-related quality of life and emotion dysregulation. Supportive therapy participants experienced reductions in a broader range of self-destructive behaviours (eg, substance misuse, impulsive sex), not seen in SAFETY.

Drop-out and attrition were low, with only 1 participant (3%) dropping out of treatment and 28 (93%) participants having complete outcome data at 3-month post-treatment. The numbers of completed sessions were similar across conditions, and the proportions who received the full treatment were comparable to the findings from the US SAFETY RCT^11^ and the US DBT-trial,^6^ which included supportive therapy as the control condition. The flexible treatment format of SAFETY^11 19^—which allows therapists to conduct treatment sessions via online video calls, in-home or in inpatient settings as needed—likely improved treatment compliance.^20^ In SAFETY, both youths and parents reported high treatment satisfaction. While acceptable, both youths and parents in supportive therapy reported lower satisfaction. Given that parental involvement may be a key component of treatments targeting suicide attempt and NSSI in youth,^21^ the finding that both youths and parents actively engaged in and reported high satisfaction with SAFETY is important. To further highlight these results—what they suggest is that parents are willing to participate in the treatment together with their youth, and that both youths and parents reported satisfaction with a treatment delivered in a family format.

Large reductions in NSSI were observed for SAFETY participants. Recent advancements in psychosocial treatment for NSSI emphasise the importance of targeting emotion dysregulation.^10 12^ Indeed, large improvements in emotion dysregulation were observed in SAFETY. Adequately powered trials are needed to determine the effects of SAFETY on NSSI and to assess whether emotion dysregulation mediates the effect. Comparable to previous findings,^13^ SAFETY participants experienced decreased functional impairment and improvements in depressive symptoms. Moreover, SAFETY participants also experienced improvements in quality of life—a relevant factor in recovery after a suicide attempt or NSSI^22^—which had not been studied in previous SAFETY trials.^11 13^ The quality of life measure CHU-9D^23^ used in this trial can be used to calculate quality-adjusted life-year, a measure of disease burden used in cost-effectiveness studies, making it a promising outcome for future trials. Decreased hopelessness was observed in both conditions; however, parents showed small, non-significant increases. This may reflect parents learning previously hidden details about their youth’s mental health and the seriousness of suicidal behaviour from therapists and healthcare providers. Parents in both conditions showed reduced dismissal of their child’s emotions, aligning with the treatments’ focus on validation.^6 11^ These findings suggest parental involvement may influence intended behaviour changes, though links to youth emotion regulation or suicide attempts require future study.

### Strengths and limitations

This feasibility trial had several strengths. These include the randomised design, use of the active control condition and the clinically referred sample. The exclusion criteria were kept to a bare minimum, improving external validity of the findings. Assessments included both clinician-administered and self-rated instruments, with reports from both parent and youth, and there was little missing data. The study also had limitations. The current study was embedded within CAMHS and all participants were clinically referred to the study. The CAMHS referral portal and clinicians were instructed to refer all youths with recent suicidal behaviour to the study. We believe this recommendation was generally followed and that the reasons for exclusion described in figure 1 are representative. However, given that the extent of prescreening is not known, this might affect the generalisability of findings. Based on ethical and practical considerations, participants were allowed to receive other concurrent treatments during the trial, and stability in psychopharmacological medication was not required for study inclusion. However, parallel treatment was measured and found to be comparable across groups. Furthermore, the sample was predominantly female. While suicide attempts are more common among females, males die by suicide more often.^3^ Future studies should strive towards recruiting a more gender-balanced sample.

## Clinical implications

The results of this feasibility trial support the feasibility and acceptability of the two evaluated treatments (SAFETY, supportive therapy). A full-scale RCT should include a large sample size, taking the possibility of small effect sizes into account, to establish the efficacy of SAFETY for youth with suicidal behaviour and NSSI.

## Supplementary material

10.1136/bmjment-2025-301575 online supplemental file 1

## Data Availability

Data are available upon reasonable request.
