# Supplementary material for: Preventing suicide with Safe Alternatives for Teens and Youths (SAFETY): a randomised feasibility trial
Source: BMJ Ment Health. 2025 Apr 29;28(1):e301575. doi: 10.1136/bmjment-2025-301575 (PMC12049871; doi:10.1136/bmjment-2025-301575)
Supplement: online supplemental file 1 [file bmjment-28-1-s001.docx]

**Supplement 1**

**TRIAL PROTOCOL**

Preventing suicide with Safe Alternatives for Teens and Youths (SAFETY) – A randomized feasibility trial

| Trial ID: | NCT05537623 (ClinicalTrials.gov)  2022-02825-01 (the Swedish Ethical Review Authority) |
| --- | --- |
| Version number: | 7 |
| Date: | 07-01-2025 |
| Sponsor: | Region Stockholm |
| Sponsor representative: | Göran Rydén |
| Co-sponsor: | Karolinska institutet |
| Co-sponsor representative: | Johan Bjureberg |

**Table Of Contents**

[Revision history 3](#_Toc195618034)

[Background 6](#_Toc195618035)

[Trial objectives 7](#_Toc195618036)

[Primary objective 7](#_Toc195618037)

[Secondary objectives 7](#_Toc195618038)

[Endpoints 7](#_Toc195618039)

[Research questions 7](#_Toc195618040)

[Trial design and procedures 8](#_Toc195618041)

[Overall trial design 8](#_Toc195618042)

[Procedures and flow chart 8](#_Toc195618043)

[Start and end of the clinical trial 9](#_Toc195618044)

[Subject selection 9](#_Toc195618045)

[Trial treatments 10](#_Toc195618046)

[The active treatment 10](#_Toc195618047)

[The active control treatment 10](#_Toc195618048)

[Randomisation 10](#_Toc195618049)

[Blinding 11](#_Toc195618050)

[Concomitant use of other medicinal products and treatments 11](#_Toc195618051)

[Measures 11](#_Toc195618052)

[Feasibility outcomes 11](#_Toc195618053)

[Exploratory secondary outcomes 11](#_Toc195618054)

[Handling of adverse events 14](#_Toc195618055)

[Assessment of Adverse Events (AE) 15](#_Toc195618056)

[Ethics ………………………………………………………………………………...15](#_Toc195618057)

[Statistical analyses 15](#_Toc195618058)

[Sample size calculations 16](#_Toc195618059)

[Collection, handling, and archiving of data 16](#_Toc195618060)

[References 18](#_Toc195618061)

## Revision history

| **Protocol version** | **Date of Issue** | **Summary of changes** |
| --- | --- | --- |
| 1 | 21-06-2022 | Initial study protocol. Decided to lower the sample size to 30 participants from the ethics committee approved number of n = 40. |
| 2 | 10-08-2022 | Decided to add parental ratings of the Systemic Clinical Outcome and Routine Evaluation index of family functioning and change to measure family function from a parental perspective |
| 3 | 10-12-2022 | Decided to only administer the 16-item version of the Difficulties in Emotion Regulation Scale instead of both the brief and 36-item version, all time-points  Decided to administer the short version of the Cognitive Emotion Regulation Questionnaire instead of the 36-item version, all time-points  Decided to not administer the Dialectical Behavior Therapy - Ways of Coping Checklist |
| 4 | 24-10-2022 | Included 1-tem from Credibility/Expectancy Questionnaire in weekly measures |
| 5 | 30-05-2024 | Decided to not analyze and report in the main outcome paper:   - All weekly measures, including: the Columbia Suicide Severity Rating Scale; Deliberate Self Harm Inventory – Youth version; Difficulties in Emotion Regulation Scale-16 item; Multidimensional Scale of Perceived Social Support; Systemic Clinical Outcome and Routine Evaluation index of family functioning and change; Borderline Symptom List – Supplement; 1-tem from Credibility/Expectancy Questionnaire - 12-month post-treatment assessments - Parental ratings of Credibility/Expectancy Questionnaire - Parental ratings of the Trimbos/iMTA questionnaire for Costs associated with Psychiatric illness - Youth ratings of the Pittsburgh Sleep Quality Index - Qualitative interviews with youth and caregivers   For the reason of the specific relevance of proportion of suicide attempts regarding feasibility, decided to analyze completed suicide attempts rather than the broader construct of suicidal behavior as an exploratory secondary outcome. |
| 6 | 10-11-2024 | Due to a change in local regulations, Karolinska Institutet is added as a co-sponsor. |
| 7 | 07-01-2025 | For reasons of brevity and space limitations, decided to not report in the main outcome paper:   - Additional targets mechanisms, including youth and parent ratings of the Multidimensional Scale of Perceived Social Support and the Systemic Clinical Outcome and Routine Evaluation - 15 item version, and youth ratings of the Behavioral Emotion Regulation Questionnaire, the Cognitive Emotion Regulation Questionnaire, the Emotion Beliefs Questionnaire, the Perth Alexithymia Questionnaire, as well as the Perceived Criticism Measure – 1 item version - Youth ratings of CRAFFT - Parental ratings of Difficulties in Emotion Regulation Scale-16 item version |

**Contact information**

| **Responsibility in the clinical trial** |  |
| --- | --- |
| Sponsor representative | Göran Rydén, operations manager  Child and Adolescent Psychiatry, Region Stockholm  Sachsgatan 10 118 61 Stockholm Sweden  +46-8-514 520 88 goran.ryden@regionstockholm.se |
| Co-sponsor representative | Johan Bjureberg, docent, research group leader and PI  Centre for Psychiatry Research, Department of Clinical Neuroscience, Karolinska Institutet & Region Stockholm  Norra Stationsgatan 69,  113 64 Stockholm Sweden  +46 73-091 71 12  johan.bjureberg@ki.se |
| Principal Investigator | Johan Bjureberg |
| Specify organizations involved: | Child and Adolescent Psychiatry, Region Stockholm  Centre for Psychiatry Research, Karolinska Institutet & Region Stockholm. |

## Background

Suicide is the leading cause of death in the age span 10-19 years in Sweden.^1^ The two most robust predictors of future suicide attempts are prior suicide attempts and self-harm; prior suicide attempts also predict suicide death.^2^ A large study with British youths demonstrated a lifetime prevalence of suicide attempt at 6,8%.^3^ Further, suicide attempts and self-harm among youths are associated with lower global functioning, more frequent use of clinical care, more psychosocial problems, more comorbid mental disorders, and higher risk of adverse outcomes in adulthood compared to clinical controls.^4,5^

Consequently, treating both suicide attempts and self-harm early is an urgent matter. Advances are being made within the field.^6,7^ Yet, dialectical behavior therapy (DBT) is still the only treatment with replicated demonstrated significant effect on reducing suicide attempts and self-harm among youths.^8,9^ However, DBT, with its four modes including weekly (a) individual therapy, (b) group skills training, (c) consultation team for therapists, and (d) therapist availability for phone coaching between sessions, delivered over the course of at least four to six months, is a highly intensive and time-consuming treatment. Further, DBT is generally offered to suicidal youths also presenting with symptoms of borderline personality disorder.^8,9^ Since suicide attempts occur among individuals with a range of different clinical presentations,^10^ there is an urgent need for wider options of treatment interventions. Briefer and more easily accessed treatments are essential to be able to reach and offer evidence-based care to all youths at risk.^6^ Leading institutions have suggested that progress within mental health research requires a shift away from a focus on specific mental disorders to a more transdiagnostic perspective, addressing underlying processes.^11^

One transdiagnostic mechanisms that have been proposed to play an important role in youth suicide attempts is emotion regulation.^12^ Further, emotion regulation has also been shown to mediate the effect of psychological treatments on self-harm.^13,14^ Emotion regulation can be targeted through skills training, a therapeutic component that together with an emphasis on protective social support show promise across interventions targeting suicide attempts and self-harm.^6^ One treatment that specifically addresses emotion regulation and social support capabilities is Safe Alternatives for Teens and Youths (SAFETY).^15^ SAFETY is a transdiagnostic cognitive behavioral DBT-informed family treatment, delivered across 12 weeks, designed to address the gap between availability and demand of effective treatments targeting suicide attempts and self-harm. Working directly with both youth and parents, SAFETY incorporates skills training focused on emotion regulation and distress tolerance, as well as modules directed towards building and strengthening family and social support. Participation in SAFETY has been shown to be associated with statistically significant effects on suicide attempts, depression, hopelessness, social adjustment, emergency department (ED) visits and hospitalizations, as well as on parental depression,^15,16^ demonstrated in an open pilot trial,^16^ and in a RCT comparing SAFETY to enhanced treatment as usual.^15^ Further, SAFETY is a principle driven, highly adaptable and flexible treatment that is especially suitable for the heterogeneous high-risk group of youths presenting to the child and adolescent mental health services (CAMHS) after a suicide attempt.

The present pilot study is conducted as a preparation for a full-scale RCT evaluating the effect of SAFETY compared to supportive therapy on youth suicide suicidal behavior in a RCT within a clinical setting. The pilot study will assess and ensure feasibility, acceptability, and safety of study design, procedure, treatments, and data collection. The results and learning from this pilot study will form the basis of an adequately powered RCT.

## Trial objectives

The overall objective of the current study is to build an evidence base for a scalable suicide prevention program called SAFETY for youths seeking treatment after an episode of suicidal behavior.

In a pilot RCT, we will examine the feasibility of SAFETY and supportive therapy at post-treatment and 3 months after treatment for youth with suicidal behavior. Evaluations of feasibility, acceptability, and safety based on data from this pilot study will guide and inform the design of the full-scale RCT. This approach enables the research group to make changes in the procedure for data collection, design, clinical routines, patient safety measures, treatment protocols, and assessments before initiating the full-scale RCT.

### Primary objective

The primary objective of this trial is to assess feasibility of SAFETY and study protocols.

### Secondary objectives

Secondary objectives are to investigate the preliminary effectiveness on clinical outcomes and target mechanisms.

### Endpoints

The primary endpoint of this trial is 3-month post-treatment. The secondary endpoint of this trial is post-treatment.

### Research questions

1. Is it feasible (operationalized as participation in treatment, completed assessments, reported adverse events) to offer SAFETY and Supportive Therapy, respectively, to youths with suicidal behavior and their caregivers?
2. Is the planned procedure and design (recruitment, blinded assessors, randomization and data attrition) of the study feasible?
3. What are the proportions of suicide attempt at 3-month post-treatment in the group randomized to SAFETY group and the group randomized to Supportive Therapy, respectively?
4. Is participation in SAFETY and Supportive Therapy, respectively, associated with improvements in nonsuicidal self-injury, depression, anxiety, emotion dysregulation, global function and other outcomes, and how large are the effects?

## Trial design and procedures

### Overall trial design

The trial is a single-blind parallel group randomized controlled feasibility trial, comparing the experimental treatment SAFETY with the active control treatment Supportive Therapy. Trial participation starts at randomization and ends at 12-month post-treatment. Participation includes a 12 week treatment phase, a post-treatment assessment after finishing treatment, and follow-ups at 3-month post-treatment as well as 12-month post-treatment. Thus the total length of participation is 15 months.

### Procedures and flow chart

**

###

### Start and end of the clinical trial

The randomization of the first participant marks the start of the trial. The end of the trial is defined as when the last participant completes assessments at 3-month post-treatment.

###

### Subject selection

Participants are recruited through referrals from within the Child and Adolescent Mental Health Services in Stockholm County, Sweden.

***Inclusion criteria***

To be included in the trial, subjects must meet all of the following criteria:

- Suicidal behavior (defined as suicide attempt, interrupted suicide attempt, aborted suicide attempt, or preparatory behavior) in the last 3 months
- Age 10-17 years
- At least one primary caregiver willing to participate in treatment

***Exclusion criteria***

Subjects must not be included in this trial if any of the following criteria are met:

- Symptoms requiring other immediate treatment (e.g. psychosis, severe anorexia nervosa)
- Ongoing treatment with DBT
- Individual or life circumstances that could complicate or make treatment participation impossible, or that require immediate intervention (e.g., violence in close relationships; intellectual disability)
- Insufficient understanding of the Swedish language

***Screening and inclusion***

Subject eligibility (that subjects fulfil all inclusion criteria and do not meet any exclusion criteria) is established before youths and parents sign informed consent. After informed consent is signed, youths and parents complete self-rated baseline assessments. After the baseline assessments are complete, participants are randomized. Randomization marks study inclusion.

***Withdrawal criteria***

Participants can discontinue their participation in the study at any time.

## Trial treatments

### The active treatment

SAFETY is a transdiagnostic cognitive-behavioral family treatment informed by Dialectical Behavior Therapy (DBT). The twelve week long treatment is principle based, structured in phases, and individually tailored based on a cognitive-behavioral fit analysis that specifies key risk and protective processes. Each session contains one individual component for youth and parents respectively, and one family component where youth and parents work together with therapists to practice skills identified as critical for preventing future suicidal behavior. Treatment targets are arranged in a SAFETY Pyramid, consisting of (a) safe settings; (b) safe people; (c) safe activities and actions; (d) safe thought; and (e) safe stress reactions, emphasizing strengthening protective support and validation within the family and/or social environment surrounding the youth.

### The active control treatment

Supportive Therapy is a manualized client-centered therapy. The Supportive Therapy will be adapted to match SAFETY to control for nonspecific treatment factors such as therapist characteristics, time, and treatment exposure. The Supportive Therapy program consists of twelve weekly individual sessions with the youth, focusing on the therapeutic supporting relationship between the therapist and the youth, and follow-ups with parents. Therapeutic strategies include acceptance and validation, to increase feelings of connectedness and belonging and counteract thwarted belongingness, helplessness, and hopelessness. Cognitive-behavioral techniques (e.g., active modeling, problem-solving training, cognitive restructuring) are not allowed.

### Randomisation

The randomization sequence is generated using an online tool ([www.random.org](http://www.random.org)). An independent researcher places the sequence in sealed, opaque envelopes. Subjects are included and randomized consecutively as they are found to be eligible for inclusion in the trial and have completed baseline assessments. A dedicated member of the research group not directly involved in the trial (not PI or study coordinator) handles the randomization (opening of the sealed envelopes, entering details of randomization outcome into code key) and informs recruitment personnel and therapists of randomization number. If a subject discontinues their participation, the subject’s trial-specific participant ID code will not be reused and the subject will not be allowed to re-enter the trial again.

###

### Blinding

Masked assessors are blind to treatment allocation. Participants are instructed to not disclose their treatment allocation to the masked assessor. Blinding integrity will be evaluated through comparing masked assessors guesses of treatment allocation to chance. Masked assessors are also asked to disclose whether the participant or his/her caregiver accidentally disclosed their treatment allocation during assessments.

## Concomitant use of other medicinal products and treatments

For ethical reasons, participants will be included in the trial even if they are at time of inclusion receiving ongoing psychological or pharmacological treatment. At post-treatment, all participants will be referred to continued care based on assessed individual needs.

## Measures

### Feasibility outcomes

1. Treatment compliance, defined as proportion of participants who received six treatment sessions (approximately half the treatment) and twelve treatment sessions (approximately the full treatment).
2. Assessment compliance, defined as proportion of participants who completed primary endpoint assessments
3. Presence of study-related adverse events, evaluated by a child and adolescent psychiatrist following Good Clinical Practice
4. Treatment credibility, assessed with the Credibility/Expectancy Questionnaire^17^
5. Teatment satisfaction, assessed with the Client Satisfaction Questionnaire (CSQ)^18^

### Exploratory secondary outcomes

***Clinical outcomes***

1. Suicide attempt, assessed using the masked assessor-rated Columbia Suicide Severity Rating Scale (C-SSRS)^19^
2. NSSI, assessed using the masked assessor-rated Deliberate Self-Harm Inventory – Youth version (DSHI-Y)^20^
3. Symptom severity, assessed using the masked assessor-rated Clinical Global Impression – Severity (CGI-S)^21^
4. Symptom improvement, assessed using the masked assessor-rated Clinical Global Impression – Improvement (CGI-I)^21^
5. Global function, assessed using the masked assessor-rated Children’s Global Assessment Scale (CGAS)^22^
6. Impaired functioning, assessed with the youth- and parent-rated Work and Social Adjustment Scale – Youth version (WSAS-Y)^23^
7. Health-related quality of life, assessed with the youth-rated Child Health Utility – 9D (CHU-9D)^24^
8. Depression and anxiety, assessed with the youth-rated Revised Children's Anxiety and Depression Scale (RCADS)^25^
9. Other self-destructive behaviors, assessed with the youth-rated Borderline Symptom List – Behavior Supplement (BSL-Supplement)^26^
10. Alcohol and substance misuse, assessed with the youth-rated CRAFFT^27^

***Targets mechanisms***

1. Emotion dysregulation, assessed with the youth-rated Difficulties in Emotion Regulation Scale - brief version (DERS-16)^28^
2. Hopelessness, assessed with the youth-rated Beck’s Hopelessness Scale (BHS)^29^
3. Behavioral emotion regulation, assessed with the youth-rated Behavioral Emotion Regulation Questionnaire (BERQ)^30^
4. Cognitive emotion regulation, assessed with the youth-rated Cognitive Emotion Regulation Questionnaire (CERQ)^31^
5. Emotion awareness, assessed with the youth-rated Emotion Beliefs Questionnaire (EBQ)^32^
6. Alexithymia, assessed with the youth-rated Perth Alexithymia Questionnaire (PAQ)^33^
7. Expressed emotion, assessed with the youth-rated 1-item version of the Perceived Criticism Measure (PCM)^34^
8. Perceived social support, assessed with the youth- and parent-rated Multidimensional Scale of Perceived Social Support (MSPSS)^35^
9. Family functioning, assessed with the youth- and parent-rated Systemic Clinical Outcome and Routine Evaluation index of family functioning and change (SCORE-15)^36^

***Parental outcomes***

1. Parental depression, assessed with the parent-rated Patient Health Questionnaire – 9 (PHQ-9)^37^
2. Parental anxiety, assessed with the parent-rated Generalized Anxiety Disorder 7-item Scale (GAD-7)^38^
3. Parental hopelessness, assessed with the parent-rated BHS^29^
4. Parents ability to cope with their children’s emotions, assessed with the parent-rated Coping with Children’s Negative Emotions Scale (CCNES)^39^
5. Parental emotion dysregulation, assessed with the parent-rated DERS-16^28^

***Flowchart of assessments and time-points***

| **Procedure/ measure** | **Eligibility assessment (clinician-rated)** | **Pre-treatment/ week 0** | **Weekly during treatment** | **Post-treatment** | **3-month  post-treatment** |
| --- | --- | --- | --- | --- | --- |
| Incl/exclusion criteria | X | X |  |  |  |
| Informed consent | X |  |  |  |  |
| **Diagnostic interviews** |  |  |  |  |  |
| Psychiatric disorders (MINI KID) | X |  |  |  |  |
| Borderline personality disorder criteria (SCID-II-BPD) | X |  |  |  |  |
| **Safety monitoring** |  |  |  |  |  |
| Adverse events (SAE & AE) |  |  | X | X |  |
| **Self-rated feasibility measures** |  |  |  |  |  |
| Treatment credibility/expectancy (CEQ) |  |  | X (full scale week 1, 1 item weekly) |  |  |
| Treatment satisfaction (CSQ) |  |  |  | X |  |
| Adverse events |  |  |  | X |  |
| **Masked assessor-rated** |  |  |  |  |  |
| Suicide attempt (C-SSRS) | X |  |  | X | X |
| NSSI (DSHI-Y) | X |  |  | X | X |
| Symptom severity (CGI-S) | X |  |  | X | X |
| Symptom improvement (CGI-I) |  |  |  | X | X |
| Global functioning (CGAS) | X |  |  | X | X |
| **Self-rated** |  |  |  |  |  |
| Impaired functioning (WSAS-Y) |  | X |  | X | X |
| Health-related quality of life (CHU-9D) |  | X |  | X | X |
| Suicidal behavior (C-SSRS) |  |  | X |  |  |
| NSSI (DSHI-Y) |  |  | X |  |  |
| Anxiety and depression (RCADS) |  | X |  | X | X |
| Self-destructive behaviors (BSL-Supplement) |  | X | X | X | X |
| Alcohol and substance misuse (CRAFFT) |  | X |  | X | X |
| Emotion dysregulation (DERS-16) |  | X | X | X | X |
| Perceived social support (MSPSS) |  | X | X | X | X |
| Hopelessness (BHS) |  | X |  | X | X |
| Behavioral emotion regulation (BERQ) |  | X |  | X | X |
| Cognitive emotion regulation (CERQ) |  | X |  | X | X |
| Emotion awareness (EBQ) |  | X |  | X | X |
| Alexithymia (PAQ) |  | X |  | X | X |
| Expressed emotion (PCM-1) |  | X |  | X | X |
| Family function (SCORE-15) |  | X | X | X | X |
| **Parent-rated** |  |  |  |  |  |
| Parental depression (PHQ-9) |  | X |  | X | X |
| Parental anxiety (GAD-7) |  | X |  | X | X |
| Parental hopelessness (BHS) |  | X |  | X | X |
| Parental coping with childrens’ negative emotions (CCNES) |  | X |  | X | X |
| Parental emotion dysregulation |  | X |  | X | X |
| Family function |  | X |  | X | X |
| Credibility/Expectancy Questionnaire (CEQ) |  | X |  |  |  |
| Client Satisfaction Questionnaire (CSQ) |  |  |  | X |  |

## Handling of adverse events

An adverse event (AE) is defined as any untoward medical occurrence in a subject to whom a medicinal product is administered and which does not necessarily have a causal relationship with this treatment.

A serious adverse event (SAE) is defined as any untoward medical occurrence that at any dose requires inpatient hospitalization or prolongation of existing hospitalization, results in persistent or significant disability or incapacity, results in a congenital anomaly or birth defect, is life-threatening, or results in death.

Medical and scientific assessment will be made to determine if an event is serious.

### Assessment of Adverse Events (AE)

The principal investigator is responsible for determining whether an AE is an SAE. A child and adolescent psychiatrist is responsible for determining whether there is a causal relationship between the AE/SAE and the study procedures or treatments investigated, following Good Clinical Practice. Clinicians are recording AEs occurring during the study period continuously. For each record of AE/SAE, a description of the incident, date, severity, causal relationship, whether it is an AE or SAE, as well as an action plan and when executed a description of how the AE was dealt with is included. Any SAEs will be reported to the sponsor within 24 hours through the local system for reporting AEs within the CAMHS.

## Ethics

The trial was approved by the Swedish Ethical Review Authority (ref. no.: 2022-02825-01). The trial will be executed in compliance with this study protocol, the Declaration of Helsinki and ICH-GCP (Good Clinical Practice).

## Statistical analyses

Descriptive statistics will be calculated for all demographic and baseline characteristics, as well as for feasibility outcomes and the secondary clinical outcome suicide attempt (C-SSRS).

All analyses will be conducted according to the intention-to-treat principle, including all participants as randomized.

Within-group analyses of treatment effects on secondary outcomes will be conducted. All models will test effects from pre-treatment to post-treatment as well as from pre-treatment to 3-month post-treatment.

Different models will be used for count, ordinal, and linear outcomes. For count outcomes, model fit will be tested comparing a zero-inflated negative binomial generalized linear mixed-effects regression model, a zero-inflated Poisson regression model, and a Poisson regression model. Effect sizes for count outcomes will be presented as incidence rate ratios (IRR) with confidence intervals (CI) between time-points for the separate conditions.

Ordinal outcomes will be analyzed with linear quantile mixed regression models, using the lqmm package in R.^40–42^ Effect sizes will be calculated by dividing the unstandardized betas with the inter quartile range at the first time point, and bootstrap (1000 simulations) CI will be calculated using the functions implemented in the lqmm package.

Continuous outcomes will be analyzed with linear mixed effects regression models. Effect sizes will be evaluated with Cohen’s *d* for mixed effects models (calculated by dividing the unstandardized betas by the standard deviation at the first time point) with bootstrap (1000 simulations) CI.^43^

All models will be fitted separately for each treatment condition and include a random intercept and the dummy coded time variable (pre-treatment and post-treatment, or pre-treatment and 3-month post-treatment).

Presence of, and changes in, psychopharmacological medication from pre- to post-treatment and from post-treatment to 3-month post-treatment will be calculated and presented with descriptive statistics.

Masked assessors’ treatment allocation guesses at post-treatment and 3-month post-treatment will be analyzed with a binomial test.

Statistical analyses will be conducted using R (version 4.3.1).^42^ All tests will be 2-sided and statistical significance will be set at *P* < .05.

### Sample size calculations

Following recommendations from the Consort 2010 Statement extension for feasibility randomized controlled trials,^44^ the sample size of 30 participants was deemed suitable for assessing feasibility in this RCT.

## Collection, handling, and archiving of data

All individuals handling personal data are bound by confidentiality agreements and/or professional secrecy obligations through their employment terms or confidentiality agreements in accordance with the sponsor. Each study participant's personal data will be protected by confidentiality, and participants will be given a number as their identifier. All correspondence between participants and therapists will take place on platforms which require two-factor authentication. The code key is stored in a security-classified database for sensitive personal data within the organization of the sponsor. Data is stored in accordance with the sponsors archival rules for research documents.

Assessments are administered via the internet and all answers, from participants and clinicians, are securely stored on a database developed for this purpose and have been used in several completed and ongoing studies. All data traffic is encrypted, ensuring a high security level.

## References

1. World Health Organization (WHO). Global health estimates: Leading causes of death [Internet]. 2020 [cited 2021 Dec 10]. Available from: https://www.who.int/data/maternal-newborn-child-adolescent-ageing/advisory-groups/gama/gama-related-resources/gho

2. Ribeiro JD, Franklin JC, Fox KR, Bentley KH, Kleiman EM, Chang BP, et al. Self-injurious thoughts and behaviors as risk factors for future suicide ideation, attempts, and death: a meta-analysis of longitudinal studies. Psychol Med. 2016 Jan;46(2):225–36.

3. Mars B, Heron J, Klonsky ED, Moran P, O’Connor RC, Tilling K, et al. What distinguishes adolescents with suicidal thoughts from those who have attempted suicide? A population-based birth cohort study. J Child Psychol Psychiatry. 2019 Jan;60(1):91–9.

4. Bjureberg J, Ohlis A, Ljótsson B, D’Onofrio BM, Hedman-Lagerlöf E, Jokinen J, et al. Adolescent self-harm with and without suicidality: cross-sectional and longitudinal analyses of a Swedish regional register. J Child Psychol Psychiatry. 2019 Mar;60(3):295–304.

5. Bjureberg J, Kuja-Halkola R, Ohlis A, Lichtenstein P, D’Onofrio BM, Hellner C, et al. Adverse clinical outcomes among youths with nonsuicidal self-injury and suicide attempts: a longitudinal cohort study. J Child Psychol Psychiatry. 2021 Dec 2;

6. Glenn CR, Esposito EC, Porter AC, Robinson DJ. Evidence Base Update of Psychosocial Treatments for Self-Injurious Thoughts and Behaviors in Youth. J Clin Child Adolesc Psychol. 2019 Jun;48(3):357–92.

7. Witt KG, Hetrick SE, Rajaram G, Hazell P, Taylor Salisbury TL, Townsend E, et al. Interventions for self-harm in children and adolescents. Cochrane Database Syst Rev. 2021 Mar 7;3(3):CD013667.

8. McCauley E, Berk MS, Asarnow JR, Adrian M, Cohen J, Korslund K, et al. Efficacy of Dialectical Behavior Therapy for Adolescents at High Risk for Suicide: A Randomized Clinical Trial. JAMA Psychiatry. 2018 Aug 1;75(8):777–85.

9. Mehlum L, Tørmoen AJ, Ramberg M, Haga E, Diep LM, Laberg S, et al. Dialectical behavior therapy for adolescents with repeated suicidal and self-harming behavior: a randomized trial. J Am Acad Child Adolesc Psychiatry. 2014 Oct;53(10):1082–91.

10. Van Orden KA, Witte TK, Cukrowicz KC, Braithwaite SR, Selby EA, Joiner TE. The interpersonal theory of suicide. Psychol Rev. 2010 Apr;117(2):575–600.

11. Insel T, Cuthbert B, Garvey M, Heinssen R, Pine DS, Quinn K, et al. Research domain criteria (RDoC): toward a new classification framework for research on mental disorders. Am J Psychiatry. 2010 Jul;167(7):748–51.

12. Gratz KL, Spitzen TL, Tull MT. Expanding our understanding of the relationship between nonsuicidal self-injury and suicide attempts: The roles of emotion regulation self-efficacy and the acquired capability for suicide. J Clin Psychol. 2020 Sep;76(9):1653–67.

13. Asarnow JR, Berk MS, Bedics J, Adrian M, Gallop R, Cohen J, et al. Dialectical Behavior Therapy for Suicidal Self-Harming Youth: Emotion Regulation, Mechanisms, and Mediators. J Am Acad Child Adolesc Psychiatry. 2021 Sep;60(9):1105-1115.e4.

14. Bjureberg J, Ojala O, Hesser H, Häbel H, Sahlin H, Gratz KL, et al. Effect of Internet-Delivered Emotion Regulation Individual Therapy for Adolescents With Nonsuicidal Self-Injury Disorder: A Randomized Clinical Trial. JAMA Network Open. 2023 Jul 13;6(7):e2322069.

15. Asarnow JR, Hughes JL, Babeva KN, Sugar CA. Cognitive-Behavioral Family Treatment for Suicide Attempt Prevention: A Randomized Controlled Trial. J Am Acad Child Adolesc Psychiatry. 2017 Jun;56(6):506–14.

16. Asarnow JR, Berk M, Hughes JL, Anderson NL. The SAFETY Program: a treatment-development trial of a cognitive-behavioral family treatment for adolescent suicide attempters. J Clin Child Adolesc Psychol. 2015;44(1):194–203.

17. Devilly GJ, Borkovec TD. Psychometric properties of the credibility/expectancy questionnaire. J Behav Ther Exp Psychiatry. 2000 Jun;31(2):73–86.

18. Attkisson CC, Zwick R. The client satisfaction questionnaire. Psychometric properties and correlations with service utilization and psychotherapy outcome. Eval Program Plann. 1982;5(3):233–7.

19. Posner K, Brown GK, Stanley B, Brent DA, Yershova KV, Oquendo MA, et al. The Columbia-Suicide Severity Rating Scale: initial validity and internal consistency findings from three multisite studies with adolescents and adults. Am J Psychiatry. 2011 Dec;168(12):1266–77.

20. Gratz KL, Latzman RD, Young J, Heiden LJ, Damon J, Hight T, et al. Deliberate self-harm among underserved adolescents: the moderating roles of gender, race, and school-level and association with borderline personality features. Personal Disord. 2012 Jan;3(1):39–54.

21. Guy W. Clinical global impression. In: Guy W, editor. ECDEU Assessment manual for Psychopharmacology (revised). Rockville (Md): National Institute of Mental Health; 1976. p. 217–22.

22. Shaffer D, Gould MS, Brasic J, Ambrosini P, Fisher P, Bird H, et al. A children’s global assessment scale (CGAS). Arch Gen Psychiatry. 1983 Nov;40(11):1228–31.

23. Jassi A, Lenhard F, Krebs G, Gumpert M, Jolstedt M, Andrén P, et al. The Work and Social Adjustment Scale, Youth and Parent Versions: Psychometric Evaluation of a Brief Measure of Functional Impairment in Young People. Child Psychiatry Hum Dev. 2020 Jun;51(3):453–60.

24. Stevens K. Assessing the performance of a new generic measure of health-related quality of life for children and refining it for use in health state valuation. Appl Health Econ Health Policy. 2011 May 1;9(3):157–69.

25. Chorpita BF, Yim L, Moffitt C, Umemoto LA, Francis SE. Assessment of symptoms of DSM-IV anxiety and depression in children: a revised child anxiety and depression scale. Behav Res Ther. 2000 Aug;38(8):835–55.

26. Bohus M, Limberger MF, Frank U, Sender I, Gratwohl T, Stieglitz RD. [Development of the Borderline Symptom List]. Psychother Psychosom Med Psychol. 2001 May;51(5):201–11.

27. Knight JR, Shrier LA, Bravender TD, Farrell M, Vander Bilt J, Shaffer HJ. A new brief screen for adolescent substance abuse. Arch Pediatr Adolesc Med. 1999 Jun;153(6):591–6.

28. Bjureberg J, Ljótsson B, Tull MT, Hedman E, Sahlin H, Lundh LG, et al. Development and Validation of a Brief Version of the Difficulties in Emotion Regulation Scale: The DERS-16. J Psychopathol Behav Assess. 2016 Jun;38(2):284–96.

29. Beck AT, Steer RA, Pompili M. BHS, Beck hopelessness scale: manual. San Antonio, TX: Psychological corporation; 1988. 29 p.

30. Kraaij V, Garnefski N. The behavioral emotion regulation questionnaire: development, psychometric properties and relationships with emotional problems and the cognitive emotion regulation questionnaire. Personality and Individual Differences. 2019;137:56–61.

31. Garnefski N, Kraaij V. The cognitive emotion regulation questionnaire. European journal of psychological assessment. 2007;23(3):141–9.

32. Becerra R, Preece DA, Gross JJ. Assessing beliefs about emotions: Development and validation of the Emotion Beliefs Questionnaire. PLoS One. 2020;15(4):e0231395.

33. Preece D, Becerra R, Robinson K, Dandy J, Allan A. The psychometric assessment of alexithymia: Development and validation of the Perth Alexithymia Questionnaire. Personality and Individual Differences. 2018;132:32–44.

34. Hooley JM, Teasdale JD. Predictors of relapse in unipolar depressives: expressed emotion, marital distress, and perceived criticism. J Abnorm Psychol. 1989 Aug;98(3):229–35.

35. Zimet GD, Dahlem NW, Zimet SG, Farley GK. The multidimensional scale of perceived social support. Journal of personality assessment. 1988;52(1):30–41.

36. Stratton P, Bland J, Janes E, Lask J. Developing an indicator of family function and a practicable outcome measure for systemic family and couple therapy: the SCORE: Systemic family and couple therapy. Journal of Family Therapy. 2010 Jul 14;32(3):232–58.

37. Kroenke K, Spitzer RL, Williams JB. The PHQ-9: validity of a brief depression severity measure. J Gen Intern Med. 2001 Sep;16(9):606–13.

38. Spitzer RL, Kroenke K, Williams JBW, Löwe B. A brief measure for assessing generalized anxiety disorder: the GAD-7. Arch Intern Med. 2006 May 22;166(10):1092–7.

39. Fabes RA, Eisenberg N, Bernzweig J. The Coping with Children’s Negative Emotions Scale Adolescent Version: Procedures and Scoring. Unpublished manuscript, Arizona State University. 1998;

40. Geraci M. Linear Quantile Mixed Models: The lqmm Package for Laplace Quantile Regression. Journal of Statistical Software. 2014 May 6;57:1–29.

41. Geraci M, Bottai M. Linear quantile mixed models. Stat Comput. 2014 May 1;24(3):461–79.

42. R Core Team. R: A Language and Environment for Statistical Computing [Internet]. Vienna, Austria: R Foundation for Statistical Computing; 2023. Available from: https://www.R-project.org/

43. Feingold A. New Approaches for Estimation of Effect Sizes and their Confidence Intervals for Treatment Effects from Randomized Controlled Trials. Quant Method Psychol. 2019;15(2):96–111.

44. Eldridge SM, Chan CL, Campbell MJ, Bond CM, Hopewell S, Thabane L, et al. CONSORT 2010 statement: extension to randomised pilot and feasibility trials. BMJ. 2016 Oct 24;355:i5239.

**Supplement 2**

**Supplementary Methods
and Results**Preventing suicide with Safe Alternatives for Teens and Youths (SAFETY) – A randomized feasibility trial

**Table Of Contents**

Methods 3

Outcomes 3

Therapist treatment fidelity 7

References 8

Table S1. List of Measures and Assessment Points 11

Table S2. Content of the Safe Alternatives for Teens and Youths (SAFETY) treatment 12

Table S3. Model comparisons for count outcomes 13

Table S4. Parallel psychosocial treatment 14

Table S5. Parallel psychopharmacological treatment 15

## Methods

### Outcomes

***Diagnostic assessments***

A diagnostic interview was conducted by either a licensed psychologist, a clinical psychology intern, or a social worker, using the MINI-KID International Neuropsychiatric Interview, version 6.[1] Symptoms of borderline personality disorder was assessed using the borderline personality disorder section of the Structured Clinical Interview for DSM-IV Axis II Disorders (SCID-II).[2]

***Feasibility outcomes***

Treatment satisfaction was measured using the Client Satisfaction Questionnaire (CSQ).[3] The CSQ-8 is an 8-item self-rated 4-point scale, measuring different aspects of satisfaction with treatment, e.g. perception of quality of treatment, if the treatment adequately addressed their needs and overall satisfaction. Total range is 8 to 32, with higher values indicating higher satisfaction with treatment. This measure was self-rated by youths and parents immediately post-treatment.

Treatment credibility was measured using the Credibility/Expectancy Questionnaire (CEQ).[4] An overall credibility rating was calculated by calculating the average of the first three items of the CEQ (range 3-27), following previous research.[5] Higher scores indicate greater credibility. This measure was self-rated by youths at week 1 of treatment (after the first treatment session).

***Exploratory secondary outcomes***

*Clinical outcomes*

Suicide attempt was measured with the Columbia Suicide Severity Rating Scale (C-SSRS).[6] The C-SSRS is a 22-item structured interview assessing the frequency and severity of suicidal ideation and suicidal behavior. The C-SSRS was clinician-administered before treatment and administered by masked assessors at post-treatment and 3-month post-treatment. The interrater reliability for the suicide attempt item of the C-SSRS was 100%.

Nonsuicidal self-injury was measured with the Deliberate Self-Harm Inventory - Youth version (DSHI-Y).[7] The DSHI-Y is a 7-item measure assessing occurrence, method, frequency of deliberate self-harm. A higher score indicates higher frequency and severity of self-harm. The DSHI-Y was clinician-administered before treatment and administered by masked assessors at post-treatment and 3-month post-treatment.

Symptom severity was measured with the Clinical Global Impressions - Severity scale (CGI-S).[8] The CGI-S is a clinician-rated 1-item scale ranging from 1-7 where higher ratings indicate greater symptom severity. The CGI-S was clinician-rated before treatment and rated by masked assessors at post-treatment and 3-month post-treatment.

Symptom improvement was measured with the Clinical Global Impressions - Improvement scale (CGI-I).[8] The CGI-I is a clinician-rated 1-item scale ranging from 1-7 where a lower score indicates greater symptom improvement. The CGI-I was clinician-rated before treatment and rated by masked assessors at post-treatment and 3-month post-treatment.

Global function was measured using the Children's Global Assessment Scale (CGAS).[9] The CGAS is a single item clinician-rated 1-100 scale that integrates psychological, social, and academic functioning in children as a measure of psychiatric disturbance. Higher values represent better global function. The CGAS was clinician-rated before treatment and rated by masked assessors at post-treatment and 3-month post-treatment.

Impaired functioning in different aspects of the youths’ life was measured with the Work and Social Adjustment Scale, youth version (WSAS-Y).[10] WSAS-Y consists of five items on a Likert scale ranging from 0-8. The scale generates a global score ranging from 0 to 40, with higher scores indicating greater impairment. The WSAS-Y was self-rated by youths and parents at pre-treatment, immediately post-treatment, and at 3-month post-treatment, and rated by masked assessors at post-treatment and 3-month post-treatment.

Quality of life was measured with the Child Health Utility 9D (CHU-9D).[11] The CHU-9D is a measure of health-related quality of life, with 9 items on a Likert scale ranging from 1-5. The total score ranges from 9-45, with a higher score indicating greater health-related quality of life. The CHU-9D was self-rated by youths at pre-treatment, immediately post-treatment, and at 3-month post-treatment.

Symptoms of anxiety and depression was measured with the Revised Children's Anxiety and Depression Scale – 25-item version (RCADS-25).[12] The RCADS-25 is a shortened version of the Spence Child Anxiety Scale, with 25 items on a Likert scale ranging from 0-3. The scale includes two subscales, on for depression and one for anxiety, as well as a total score. The total score ranges from 0-75, with higher values representing worse anxiety and depression symptoms. The RCADS-25 was self-rated by youths at pre-treatment, immediately post-treatment, and at 3-month post-treatment.

Past week engagement in self-destructive behaviors (e.g. substance misuse, risky sexual behavior) was measured with an adapted version of the Borderline Symptom List Supplement (BSL-Supplement).[13] This version of the BSL-Supplement has 9 items, with scores ranging from 0-36 and a higher score indicating higher frequency of self-destructive behaviors. The BSL-Supplement was self-rated by youths at pre-treatment, weekly during treatment, immediately post-treatment, and at 3-month post-treatment.

*Target mechanisms*

Emotion dysregulation was measured with the brief 16-item version of the Difficulties In Emotion Regulation Scale (DERS-16).[14] The DERS-16 is a brief 16-item version of the DERS, a measure of difficulties in emotion regulation. The items are rated on a 1-5 Likert scale, with scores ranging from 16-80 and a higher score indicating greater difficulties with emotion regulation. This measure was self-rated by youths at pre-treatment, immediately post-treatment, and at 3-month post-treatment.

Hopelessness was measured with Beck's Hopelessness Scale (BHS).[15] The BHS consists of 20 dichotomous "true/false" items. The total score ranges from 0-20. A higher score indicates greater perceived hopelessness. The BHS was self-rated by youths at pre-treatment, immediately post-treatment, and at 3-month post-treatment.

*Parental outcomes*

Parental symptoms of depression was measured with the Patient Health Questionnaire-9 (PHQ-9).[16] The PHQ-9 consists of 10 items on a Likert scale ranging from 0-3, divided into two subscales covering symptoms and disability, respectively. The total score ranges from 0-30, with a higher score indicating greater depressive symptoms. The PHQ-9 was self-rated by parents at pre-treatment, immediately post-treatment, and at 3-month post-treatment.

Parental symptoms of anxiety was measured with the Generalized Anxiety Disorder 7-item Scale (GAD-7).[17] The GAD-7 consists of 7 items on a Likert scale ranging from 0-3. The total score ranges from 0-21, with a higher score indicating greater anxiety symptoms. The GAD-7 was self-rated by parents at pre-treatment, immediately post-treatment, and at 3-month post-treatment.

Parental hopelessness was measured with the BHS.[15] The BHS was self-rated by parents at pre-treatment, immediately post-treatment, and at 3-month post-treatment.

Parental perceived coping responses in response to youths' negative emotions were measured with the Coping with Children's Negative Emotions Scale - Adolescent Version (CCNES-A).[18] The CCNES-A version used in this trial has four subscales, each measuring different types of parental coping responses including one adaptive and supportive responses and three non-supportive responses. Each subscale has 9 items and each subscale score ranges from 1-7. Lower scores indicate more effective coping responses for all subscales except the expressive encouragement subscale, for which a higher score indicates an effective coping response. The CCNES-A was self-rated by parents at pre-treatment, immediately post-treatment, and at 3-month post-treatment.

***Additional measures, administered but not reported in the primary manuscript***

Alcohol and substance use was measured with the CRAFFT.[19] CRAFFT is a screening measure with 9 items on two subscales. The total score ranges from 0-6. A higher score indicates greater difficulties related to substance use. The CRAFFT was self-rated by youths at pre-treatment, immediately post-treatment, and at 3-month post-treatment.

Sleep disturbance was measured with the Pittsburgh Sleep Quality Index (PSQI).[20] The PSQI consists of 18 items. A higher score indicates greater sleep problems. The PSQI was self-rated by youths at pre-treatment, immediately post-treatment, and at 3-month post-treatment.

Use of behavioral emotion regulation strategies was measured with the Behavioral Emotion Regulation Questionnaire (BERQ).[21] The BERQ consist of 20 items on a Likert scale ranging from 1-5. A higher score indicates greater difficulties with behavioral emotion regulation. This scale has five subscales. Each total subscale score ranges from 4-20, with higher scores indicating greater usage of a certain strategy. The BERQ was self-rated by youths at pre-treatment, immediately post-treatment, and at 3-month post-treatment.

Use of cognitive emotion regulation strategies was measured with the short 18-item version of the Cognitive Emotion Regulation Questionnaire (CERQ-18).[22] The CERQ-18 is a measure of cognitive emotion regulation, with 18 items on a Likert scale ranging from 1-5 and nine subscales that can be divided into maladaptive and adaptive cognitive strategies. For each subscale, the total score ranges from 4-20. The higher the subscale score, the more a specific cognitive strategy is used. This measure was self-rated by youths at pre-treatment, immediately post-treatment, and at 3-month post-treatment.

Emotion awareness was measured with the Emotion Beliefs Questionnaire (EBQ).[23] The EBQ consists of 16 items on a Likert scale ranging from 1-7. The total score ranges from 16-112, with a higher score indicating more maladaptive beliefs about emotions. The EBQ was self-rated by youths at pre-treatment, immediately post-treatment, and at 3-month post-treatment.

Alexithymia was measured with the Perth Alexithymia Questionnaire (PAQ).[24] The PAQ is a measure of how emotions are perceived and experienced, with 24 items on a Likert scale ranging from 1-7. A higher score indicates higher levels of alexithymia. The PAQ was self-rated by youths at pre-treatment, immediately post-treatment, and at 3-month post-treatment.

Expressed emotion was measured with the 1-item version of the Perceived Criticism Measure (PCM - 1 item version).[25] The PCM-1consists of one question on a Likert scale ranging from 0-10, about how critical an emotionally important individual is perceived to be by the rater. A higher score indicates higher perceived expressed emotion. The PCM-1 was self-rated by youths at pre-treatment, immediately post-treatment, and at 3-month post-treatment.

Perceived social support was measured with the Multidimensional Scale of Perceived Social Support (MSPSS).[26] The MSPSS consists of 12 items on a Likert scale ranging from 1-7. This scale has three subscales. The total score ranges from 12-84. A higher score indicates higher level of perceived social support. The MSPSS was self-rated by youths at pre-treatment, immediately post-treatment, and at 3-month post-treatment.

Family function was rated with the Systemic Clinical Outcome and Routine Evaluation, 15 item version (SCORE-15).[27] The SCORE-15 consists of 15 items on a Likert scale ranging from 1-5. The total score ranges from 15-72. A higher score indicates greater family function. The SCORE-15 was self-rated by youths and parents at pre-treatment, immediately post-treatment, and at 3-month post-treatment, and by parents at pre-treatment, immediately post-treatment, and at 3-month post-treatment.

Treatment expectancy was measured using the Credibility/Expectancy Questionnaire (CEQ).[4] Expectancy was measured by item four “By the end of the therapy period, how much improvement in your symptoms do you think will occur?” (range 0-100 in 10-point increments). Higher scores indicate greater expectancy. This measure was self-rated by youths at week 1 of treatment (after the first treatment session).

Parental emotion dysregulation was measured with the DERS-16.[14] This measure was self-rated by parents at pre-treatment, immediately post-treatment, and at 3-month post-treatment.

### Therapist treatment fidelity

A randomly selected sample of 10% of videotaped sessions per therapist were rated for fidelity by a SAFETY co-developer, using rating scales from previous trials of SAFETY[28] including the Cognitive Therapy Rating Scale (CTRS) and the Multi-Systemic Therapy Therapist Adherence Measure-Revised (TAM-R).[29] Sessions conducted in Swedish were transcribed, with the rater using both the recording and transcription to rate the session. Ratings indicated strong adherence to CBT principles, with 100% of sessions exceeding the CTRS adherence score of 40[30] (mean=60.0, SD=4.2), and indicated strong use of MST principles (mean = 4.2, SD = 1.5).

## References

1. Sheehan DV, Sheehan KH, Shytle RD, Janavs J, Bannon Y, Rogers JE, et al. Reliability and validity of the Mini International Neuropsychiatric Interview for Children and Adolescents (MINI-KID). J Clin Psychiatry. 2010 Mar;71(3):313–26.

2. First MB, Gibbon M, Spitzer RL, Williams JBW, Smith Benjamin L. Structured clinical interview for DSM-IV personality disorders (SCID-II): Interview and questionnaire. Washington DC: APA; 1997.

3. Attkisson CC, Zwick R. The client satisfaction questionnaire. Psychometric properties and correlations with service utilization and psychotherapy outcome. Eval Program Plann. 1982;5(3):233–7.

4. Devilly GJ, Borkovec TD. Psychometric properties of the credibility/expectancy questionnaire. J Behav Ther Exp Psychiatry. 2000 Jun;31(2):73–86.

5. Thompson-Hollands J, Bentley KH, Gallagher MW, Boswell JF, Barlow DH. Credibility and outcome expectancy in the unified protocol: Relationship to outcomes. J Exp Psychopathol. 2014;5(1):72–82.

6. Posner K, Brown GK, Stanley B, Brent DA, Yershova KV, Oquendo MA, et al. The Columbia-Suicide Severity Rating Scale: initial validity and internal consistency findings from three multisite studies with adolescents and adults. Am J Psychiatry. 2011 Dec;168(12):1266–77.

7. Gratz KL, Latzman RD, Young J, Heiden LJ, Damon J, Hight T, et al. Deliberate self-harm among underserved adolescents: the moderating roles of gender, race, and school-level and association with borderline personality features. Personal Disord. 2012 Jan;3(1):39–54.

8. Guy W. Clinical global impression. In: Guy W, editor. ECDEU Assessment manual for Psychopharmacology (revised). Rockville (Md): National Institute of Mental Health; 1976. p. 217–22.

9. Shaffer D, Gould MS, Brasic J, Ambrosini P, Fisher P, Bird H, et al. A children’s global assessment scale (CGAS). Arch Gen Psychiatry. 1983 Nov;40(11):1228–31.

10. Jassi A, Lenhard F, Krebs G, Gumpert M, Jolstedt M, Andrén P, et al. The Work and Social Adjustment Scale, Youth and Parent Versions: Psychometric Evaluation of a Brief Measure of Functional Impairment in Young People. Child Psychiatry Hum Dev. 2020 Jun;51(3):453–60.

11. Stevens K. Valuation of the Child Health Utility 9D Index. PharmacoEconomics. 2012 Aug 1;30(8):729–47.

12. Chorpita BF, Yim L, Moffitt C, Umemoto LA, Francis SE. Assessment of symptoms of DSM-IV anxiety and depression in children: a revised child anxiety and depression scale. Behav Res Ther. 2000 Aug;38(8):835–55.

13. Bohus M, Limberger MF, Frank U, Sender I, Gratwohl T, Stieglitz RD. [Development of the Borderline Symptom List]. Psychother Psychosom Med Psychol. 2001 May;51(5):201–11.

14. Bjureberg J, Ljótsson B, Tull MT, Hedman E, Sahlin H, Lundh LG, et al. Development and Validation of a Brief Version of the Difficulties in Emotion Regulation Scale: The DERS-16. J Psychopathol Behav Assess. 2016 Jun;38(2):284–96.

15. Beck AT, Steer RA, Pompili M. BHS, Beck hopelessness scale: manual. San Antonio, TX: Psychological corporation; 1988. 29 p.

16. Kroenke K, Spitzer RL, Williams JB. The PHQ-9: validity of a brief depression severity measure. J Gen Intern Med. 2001 Sep;16(9):606–13.

17. Spitzer RL, Kroenke K, Williams JBW, Löwe B. A brief measure for assessing generalized anxiety disorder: the GAD-7. Arch Intern Med. 2006 May 22;166(10):1092–7.

18. Fabes RA, Eisenberg N, Bernzweig J. The Coping with Children’s Negative Emotions Scale Adolescent Version: Procedures and Scoring. Unpubl Manuscr Ariz State Univ. 1998;

19. Knight JR, Shrier LA, Bravender TD, Farrell M, Vander Bilt J, Shaffer HJ. A new brief screen for adolescent substance abuse. Arch Pediatr Adolesc Med. 1999 Jun;153(6):591–6.

20. Buysse DJ, Reynolds CF, Monk TH, Berman SR, Kupfer DJ. The Pittsburgh Sleep Quality Index: a new instrument for psychiatric practice and research. Psychiatry Res. 1989 May;28(2):193–213.

21. Kraaij V, Garnefski N. The behavioral emotion regulation questionnaire: development, psychometric properties and relationships with emotional problems and the cognitive emotion regulation questionnaire. Personal Individ Differ. 2019;137:56–61.

22. Garnefski N, Kraaij V. Cognitive emotion regulation questionnaire–development of a short 18-item version (CERQ-short). Personal Individ Differ. 2006;41(6):1045–53.

23. Becerra R, Preece DA, Gross JJ. Assessing beliefs about emotions: Development and validation of the Emotion Beliefs Questionnaire. PloS One. 2020;15(4):e0231395.

24. Preece D, Becerra R, Robinson K, Dandy J, Allan A. The psychometric assessment of alexithymia: Development and validation of the Perth Alexithymia Questionnaire. Personal Individ Differ. 2018;132:32–44.

25. Hooley JM, Teasdale JD. Predictors of relapse in unipolar depressives: expressed emotion, marital distress, and perceived criticism. J Abnorm Psychol. 1989 Aug;98(3):229–35.

26. Zimet GD, Dahlem NW, Zimet SG, Farley GK. The multidimensional scale of perceived social support. J Pers Assess. 1988;52(1):30–41.

27. Stratton P, Bland J, Janes E, Lask J. Developing an indicator of family function and a practicable outcome measure for systemic family and couple therapy: the SCORE: Systemic family and couple therapy. J Fam Ther. 2010 Jul 14;32(3):232–58.

28. Asarnow JR, Hughes JL, Babeva KN, Sugar CA. Cognitive-Behavioral Family Treatment for Suicide Attempt Prevention: A Randomized Controlled Trial. J Am Acad Child Adolesc Psychiatry. 2017 Jun;56(6):506–14.

29. Henggeler SW. Serious Emotional Disturbance in Children and Adolescents: Multisystemic Therapy. Guilford Press; 2002. 280 p.

30. Brent D, Emslie G, Clarke G, Wagner KD, Asarnow JR, Keller M, et al. Switching to another SSRI or to venlafaxine with or without cognitive behavioral therapy for adolescents with SSRI-resistant depression: the TORDIA randomized controlled trial. JAMA. 2008 Feb 27;299(8):901–13.

## Table S1. List of Measures and Assessment Points

| **Measure** | **Pre-treatment** | **Post-treatment** | **3-month  post-treatment** |
| --- | --- | --- | --- |
| **Diagnostic Interviews** |  |  |  |
| Psychiatric disorders (MINI KID) | X |  |  |
| Borderline personality disorder criteria (SCID-II-BPD) | X |  |  |
| **Self-rated feasibility measures** |  |  |  |
| Treatment credibility/expectancy (CEQ) | X (week 1) |  |  |
| Treatment satisfaction (CSQ) |  | X |  |
| Adverse events |  | X |  |
| **Masked assessor-rated** |  |  |  |
| Suicidal behavior (C-SSRS) |  | X | X |
| NSSI (DSHI-Y) |  | X | X |
| Symptom severity (CGI-S) | X | X | X |
| Symptom improvement (CGI-I) |  | X | X |
| Global functioning (CGAS) | X | X | X |
| **Self-rated** |  |  |  |
| Impaired functioning (WSAS-Y) | X | X | X |
| Health-related quality of life (CHU-9D) | X | X | X |
| Anxiety and depression (RCADS) | X | X | X |
| Self-destructive behaviors (BSL-Supplement) | X | X | X |
| Alcohol and substance misuse (CRAFFT) | X | X | X |
| Emotion dysregulation (DERS-16) | X | X | X |
| Perceived social support (MSPSS) | X | X | X |
| Hopelessness (BHS) | X | X | X |
| Behavioral emotion regulation (BERQ) | X | X | X |
| Cognitive emotion regulation (CERQ) | X | X | X |
| Emotion awareness (EBQ) | X | X | X |
| Alexithymia (PAQ) | X | X | X |
| Expressed emotion (PCM-1) | X | X | X |
| Family function (SCORE-15) | X | X | X |
| **Parent-rated** |  |  |  |
| Parental depression (PHQ-9) | X | X | X |
| Parental anxiety (GAD-7) | X | X | X |
| Parental hopelessness (BHS) | X | X | X |
| Parental coping with childrens’ negative emotions (CCNES) | X | X | X |
| Parental emotion dysregulation (DERS-16) | X | X | X |
| Family function | X | X | X |

## Table S2. Content of the Safe Alternatives for Teens and Youths (SAFETY) treatment

| **Phase** | **Session/Week** | **Major Objectives and Focus** |
| --- | --- | --- |
| **1** |  | **Establishing safety, development of cognitive-behavioral fit analysis, and treatment plan** |
|  | **Session 1** | The first session is conducted in home. This session is dedicated to developing an initial cognitive behavioral fit analysis of suicidal behavior, to start building on familial support and monitoring, aid in lethal means restriction, develop an initial safety plan, as well as working on youth and parental commitment to treatment. |
|  | **Session 2** | Continue work with the cognitive behavioral fit-analysis. Focus on identifying risk and protective factors of suicidal behavior within multiple systems and levels within the youth, family, and close environment. Continue work with safety plan, integrating new information. |
|  | **Session 3** | Develop the initial treatment plan in collaboration with youth and caregivers based on identified individualized risk and protective factors. Treatment targets are identified and linked to specific treatment modules. |
| **2** |  | **Implement treatment plan** |
|  | **Session 4- Week 9** | Implement treatment plan, update the cognitive behavioral fit analysis and treatment plan if new information is acquired. Treatment response is monitored and treatment plan is revised as needed. Session plans might be altered to deal with safety issues, following a Dialectical Behavior Therapy target hierarchy prioritizing suicidal or self-injury-related behavior if reported, followed by issues causing treatment interference; and other targets from the cognitive behavioral fit analysis. |
| **3** |  | **Consolidate gains, relapse prevention, link to follow-up care, termination** |
|  | **Week 10-12** | Relapse prevention, address termination, and link to follow-up care as needed. |

## Table S3. Model comparisons for count outcomes

|  | **AIC** | | | **BIC** | | |
| --- | --- | --- | --- | --- | --- | --- |
|  | **Poisson** | **ZIP** | **ZINB** | **Poisson** | **ZIP** | **ZINB** |
| **Masked assessor-rated NSSI  (DSHI-Y)^a^** |  |  |  |  |  |  |
| Within-group |  |  |  |  |  |  |
| SAFETY | 633. | 573 | 300. | 636. | 577. | 304. |
| Supportive Therapy | 443. | 407. | 276. | 446. | 411. | 280. |
| **Self-rated self-destructive behaviors (BSL-Supplement)^b^** |  |  |  |  |  |  |
| SAFETY | 179. | 171. | 173. | 182. | 175. | 177. |
| Supportive Therapy | 132. | 133. | 136. | 135. | 137. | 140. |
| Abbreviations: AIC, Akaike information criterion; BIC Bayesian information criterion; BSL-Supplement, Borderline Symptom List – Supplement; DSHI-Y, Deliberate Self-Harm Inventory – Youth version; ZIP, Zero-inflated Poisson; ZINB, Zero-inflated negative-binomial  ^a^ Model comparisons were done separately for each treatment condition. | | | | | | |

## Table S4. Parallel psychosocial treatment^a^

|  | **SAFETY  (n = 15)** | | | **Supportive Therapy  (n = 15)** | | | **Total  (N= 30)** | | |
| --- | --- | --- | --- | --- | --- | --- | --- | --- | --- |
|  | **Mean (SD)** | **Min** | **Max** | **Mean (SD)** | **Min** | **Max** | **Mean (SD)** | **Min** | **Max** |
| Psychosocial treatment (no. sessions) |  |  |  |  |  |  |  |  |  |
| Supportive   Therapy | 0.53 (1.55) | 0 | 6 | 0 | 0 | 0 | 0.27 (1.11) | 0 | 6 |
| Other care contacts |  |  |  |  |  |  |  |  |  |
| Inpatient care (no.   nights) | 0.87 (3.36) | 0 | 13 | 0 | 0 | 0 | 0.43 (2.37) | 0 | 13 |
| Emergency   department visits | 0.20 (0.56) | 0 | 2 | 0.20 (0.41) | 0 | 1 | 0.20 (0.48) | 0 | 2 |
| Medical doctor   visits | 1.20 (1.32) | 0 | 4 | 0.73 (1.16) | 0 | 4 | 0.97 (1.25) | 0 | 4 |
| Assessment | 0.13 (0.35) | 0 | 1 | 0.93 (2.89) | 0 | 11 | 0.53 (2.06) | 0 | 11 |
| Other (e.g. medical   day care) | 1.80 (5.89) | 0 | 23 | 0.47 (1.06) | 0 | 4 | 1.13 (4.22) | 0 | 23 |
| ^a^ Between pre- and post-treatment. | | | | | | | | | |

## Table S5. Parallel psychopharmacological treatment

|  | **SAFETY  (n = 15)** | **Supportive Therapy  (n = 15)** | **Total  (N = 30)** |
| --- | --- | --- | --- |
|  | **n (%)** | **n (%)** | **n (%)** |
| **Use of psychopharmacological medication** | | | |
| ***Pre-treatment to post-treatment*** | | | |
| Antidepressants (N06A) |  |  |  |
| SSRI (N06AB) | 11 (73) | 9 (60) | 20 (67) |
| Other antidepressants (N06AX) | 0 (0) | 1 (7) | 1 (7) |
| Hypnotics and sedatives (N05C) |  |  |  |
| Melatonin receptor agonists (N05CH) | 4 (27) | 5 (33) | 9 (30) |
| Other hypnotics and sedatives (N05CM) | 2 (13) | 1 (7) | 3 (10) |
| Antihistamines for systemic use (R06A) | 6 (40) | 3 (20) | 9 (30) |
| Psychostimulants (N06B) | 3 (20) | 4 (27) | 7 (23) |
| Antipsychotics (N05A) | 2 (13) | 0 (0) | 2 (7) |
| Antiepileptics (N03A) | 1 (7) | 0 (0) | 1 (3) |
| ***Post-treatment to 3-month post-treatment*** | | | |
| Antidepressants (N06A) |  |  |  |
| SSRI (N06AB) | 11 (73) | 10 (67) | 21 (72) |
| Other antidepressants (N06AX) | 0 (0) | 0 (0) | 0 (0) |
| Hypnotics and sedatives (N05C) |  |  |  |
| Melatonin receptor agonists (N05CH) | 4 (27) | 2 (13) | 6 (20) |
| Other hypnotics and sedatives (N05CM) | 2 (13) | 0 (0) | 2 (7) |
| Antihistamines for systemic use (R06A) | 3 (20) | 3 (20) | 6 (20) |
| Psychostimulants (N06B) | 3 (20) | 4 (27) | 7 (23) |
| Antipsychotics (N05A) | 2 (13) | 0 (0) | 2 (7) |
| Antiepileptics (N03A) | 1 (7) | 0 (0) | 1 (3) |
| **Change in psychopharmacological medication** | | | |
| ***Pre-treatment to post-treatment*** | | | |
| Increased | 3 (20) | 2 (13) | 5 (17) |
| New | 4 (27) | 8 (53) | 12 (40) |
| Decreased | 1 (7) | 2 (13) | 3 (10) |
| Discontinued | 5 (33) | 5 (33) | 10 (33) |
| ***Post-treatment to 3-month post-treatment*** | | | |
| Increased | 0 (0) | 3 (20) | 3 (10) |
| New | 3 (20) | 3 (20) | 6 (20) |
| Decreased | 1 (7) | 2 (13) | 3 (10) |
| Discontinued | 4 (27) | 6 (40) | 10 (33) |
